# Making Sense of Heteroatom Effects in π–π Interactions

**DOI:** 10.1021/jacs.5c12769

**Published:** 2025-08-21

**Authors:** Khue U. Do, Audrey V. Conner, Steven E. Wheeler

**Affiliations:** Department of Chemistry, 138572 University of Georgia, Athens, Georgia 30602, United States

## Abstract

Heteroatoms can profoundly impact the strength and preferred geometry of parallel and T-shaped interactions between aromatic systems. Understanding these heteroatom effects is vital for the rational design of everything from pharmaceuticals to asymmetric catalysts and organic materials. We first examine how the introduction of N:, NH, and C=O groups changes the shape of the relaxed interaction energy curve for the parallel-displaced benzene dimer. These heteroatom effects are shown to be (1) additive; (2) dependent primarily on the orientation of the heteroatom(s) relative to the displacement axis; and (3) driven by electrostatic effects. We then introduce a simple conceptual model to make sense of these results based on the interaction of a local dipole associated with each heteroatom with the electric field of the other aromatic system. Finally, we demonstrate how this model can be applied to complex parallel stacked dimers as well as T-shaped interactions, providing a way to make sense of heteroatom effects in π–π interactions without computations.

## Graphical abstract

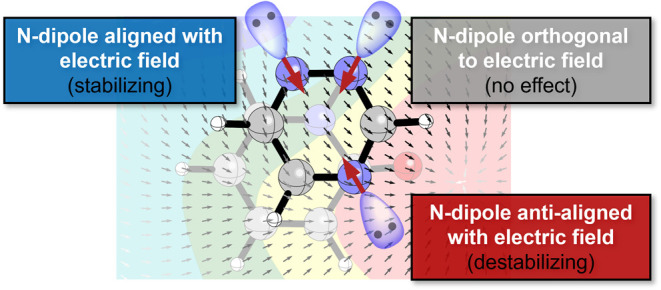

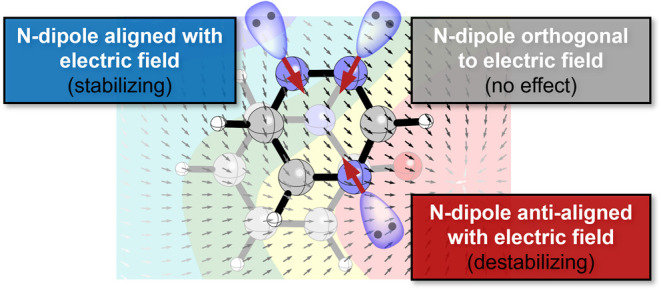

## 1 Introduction

Parallel and T-shaped interactions between aromatic molecules are ubiquitous in chemical and biological systems (e.g., see Figure 1).^1–3^ The strength and geometry of these interactions can be broadly tuned through the introduction of heteroatoms with implications for the design of everything from small molecule pharmaceuticals^4–12^ to asymmetric catalysts^13–15^ and organic materials.^16–21^ Harnessing these effects for such purposes, however, requires an understanding of how they operate.

**Figure 1:**
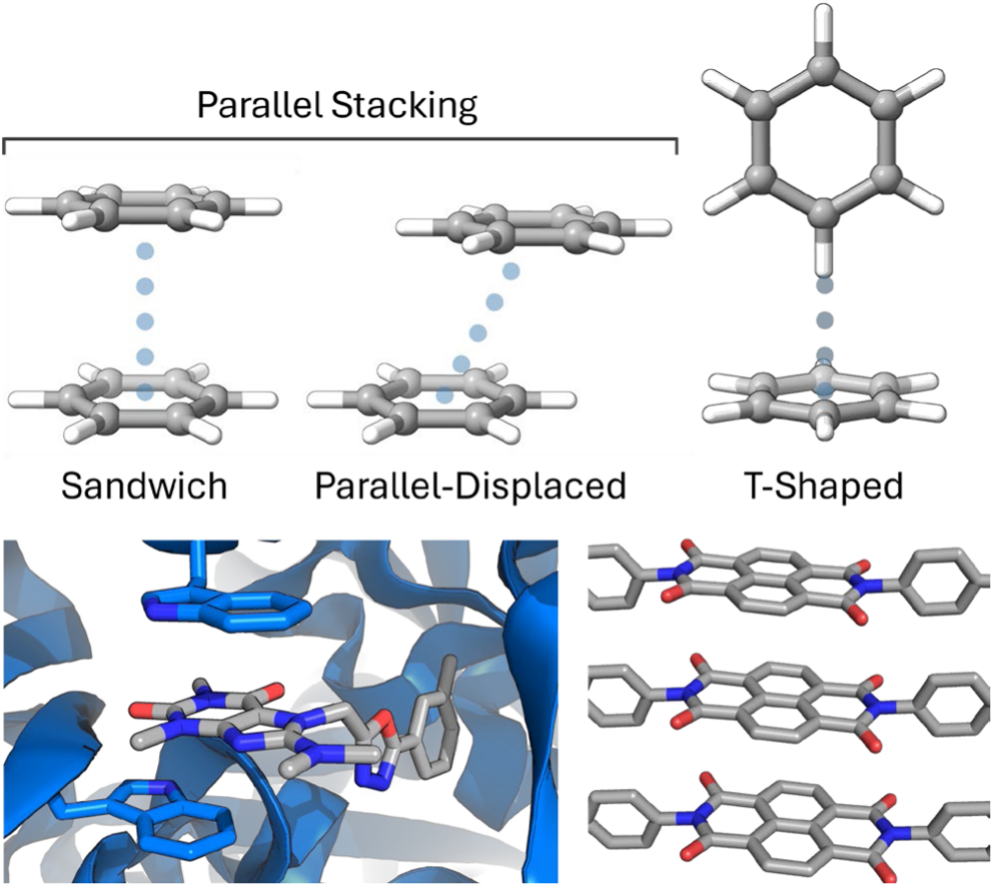
(Top) Idealized benzene dimer configurations (adapted from ref 22. Available under a CC-BY 4.0 license. Copyright Steven E. Wheeler.); (bottom left) inhibitor (gray) bound to a NUDIX hydrolase via stacking interactions with two Trp residues (PDB: 5NQR);^23^ (bottom right) molecular packing of a thermoresponsive organic material organized via stacking interactions of naphthalene diimides.^24^

Efforts to understand interactions between aromatic systems stretch back more than half a century. Early qualitative and quantitative models^25–30^ described the electrostatic component of these interactions in terms of molecular quadrupole moments. However, in 1987, Price and Stone^31^ showed that such models result in significant errors at typical stacking distances and instead advocated for the use of distributed multipoles (dipoles, quadrupoles, etc.)^32,33^ located on each (heavy) atom. Such analyses^31^ provided a means of understanding heteroatom effects on stacking interactions through their impact on the distributed multipoles of each monomer and the corresponding interactions of these multipoles.^31,34,35^ However, this distributed multipole approach was not amenable to qualitative application by organic chemists, precluding its routine use and widespread adoption.

In 1990, Hunter and Sanders presented a simpler, more intuitive view of stacking interactions^36^ based on an empirical potential. The electrostatic component of this potential used positive charges at each nuclear position to model the σ-framework, combined with a pair of negative charges above and below each π-atom to account for the π-electrons.^37^ The values of these charges were determined for each aromatic system based on simple quantum chemical computations and accounted for the redistribution of electron density due to the presence of things like heteroatoms. Hunter and Sanders also provided^36^ a set of “Rules” that, among other things, predict how heteroatoms will change the strength of π–π interactions in different orientations. Central to these Rules was the idea that favorable geometries will place “electron-poor” atoms of one aromatic system near “electron-rich” atoms of the other. In practice, the Hunter-Sanders potential and Rules are substantially simpler than the distributed multipole framework espoused by Stone and co-workers.^31–33^ Despite this simplicity, predictions from both the potential and Rules from Hunter and Sanders^36^ were in general agreement with available experimental data, and this general framework emerged as the preferred means of understanding interactions among (hetero)­aromatic rings.

Unfortunately, over the ensuing decades, the phrase “Hunter-Sanders model” grew to be synonymous with a range of models that did not appear in the original work.^22,36,38^ Regardless of the name used for the model, interactions between aromatic systems are still often discussed in terms of molecular dipoles or quadrupoles, despite well-established flaws of such an approach.^31,34^ This topic was recently revisited by Herbert et al.,^38^ who showed definitively that quadrupolar electrostatics fails qualitatively in reproducing the electrostatic component of model parallel and T-shaped interactions.

We recently demonstrated^22^ that in contrast to widespread claims about failures of the “Hunter-Sanders model”,^39–58^ the original Hunter-Sanders potential and Rules^36^ faithfully describe many aspects of interactions between aromatic systems. This includes qualitatively correct predictions of the impact of heteroatoms in parallel-displaced stacking interactions. However, we also identified shortcomings.^22^ Perhaps most importantly in the present context, both the Hunter-Sanders Rules and potential^36^ are unable to predict that the introduction of nitrogen atoms stabilizes the benzene sandwich dimer and the potential struggles to accurately describe parallel-displaced dimers in which N atoms are located over another ring.^22^ The Hunter-Sanders Rules also become cumbersome when applied to dimers of complex heterocycles. In other words, while the original Hunter-Sanders Rules^36^ perform reasonably well, they are in need of an update.

There have been numerous *ab initio* studies^9,10,38,59–71^ of model heterocyclic dimers that have provided key insights. However, none of these have supplied an intuitive framework that can replace the Hunter-Sanders Rules.^36^ Most recently, Herbert et al.^38^ showed that the van der Waals (vdW)-driven preference for parallel-displaced stacking in the benzene dimer^54,56^ is minimally perturbed by heteroatoms. Instead, heteroatoms primarily affect the electrostatic component of these interactions, which then dictates the relative energies of the parallel-displaced minima. Unfortunately, Herbert et al.^38^ did not explain these electrostatic effects, instead concluding that “*rigorous quantum-mechanical calculation of the Coulomb interaction between monomer charge densities, rather than any finite-order multipolar approximation, is necessary in order to safely draw conclusions about electrostatic interactions*”. In other words, because the common practice of rationalizing stacking interactions based on molecular multipole moments does not work, the only recourse is expensive quantum-mechanical computations. This is a false dichotomy.

Below, we show that even though directly computing accurate interaction energies of heteroaromatic dimers requires expensive computations,^38,60^ capturing the change in interaction energy due to heteroatoms is relatively simple. In fact, understanding and predicting the impact of heteroatoms on parallel and T-shaped dimers can be done without computations through a simple heuristic model based on the interaction of local dipoles associated with the heteroatoms in one system with the electric field of the other.

## 2 Computational Methods

We considered dimers of the heterocycles in Figure 2 primarily in parallel stacked arrangements with benzene as a function of horizontal (*x*) and vertical (*z*) displacements. The reference orientation of each heterocycle, relative to the +*x* axis, is shown in Figure 2. We also considered dimers in which the heterocycle is rotated from this reference orientation. For instance, pyridine(60) denotes a geometry in which pyridine is rotated 60° around the *z*-axis. Interaction energies were computed using symmetry-adapted perturbation theory (SAPT) at the SAPT2+3/def2-TZVPD level.^72–74^ This method provides accurate interaction energies^22^ in terms of electrostatic (Elec), dispersion (Disp), exchange-repulsion (Exch), and induction (Ind) effects. The sum of the dispersion and exchange-repulsion terms is discussed collectively as the vdW component. Monomers were fixed at geometries optimized at the ωB97X-D/def2-TZVP level of theory^74,75^ using Gaussian16.^76^ Dipole moments were computed at the same level of theory. Structure manipulations and input file generation were done using AaronTools.^77^ Psi4 ^78^ was used for the SAPT computations and the evaluation of electrostatic potentials (ESPs) and electric fields at the ωB97X-D/def2-TZVP level of theory. ESPs are plotted from −7.5 kcal/mol (red) to +7.5 kcal/mol (blue). See Supporting Information for details.

**Figure 2:**
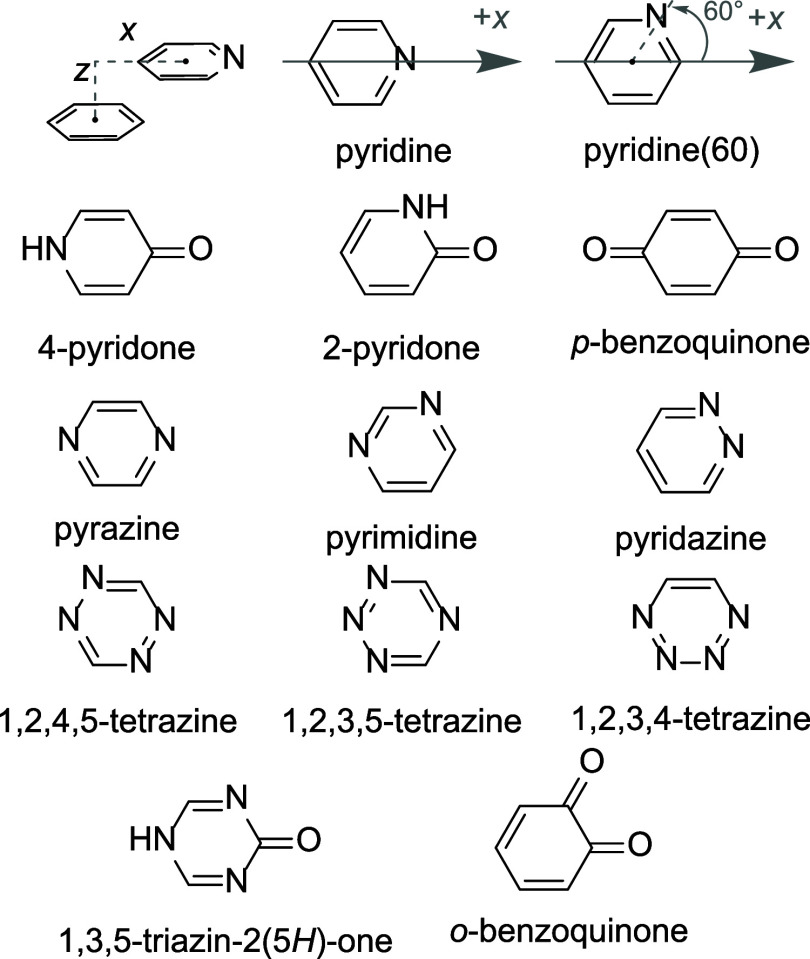
Definition of the horizontal (*x*) and vertical (*z*) displacement coordinates and heterocycles in their reference orientations relative to the +*x* axis.

## 3 Results and Discussion

### 3.1 Benzene Dimer

The interaction energy for the parallel stacked benzene dimer is plotted as a function of horizontal and vertical displacements in Figure 3. The energy along the minimum energy path (MEP), which corresponds to the stacking interaction for a given horizontal displacement at the corresponding optimal vertical separation, is also shown. This relaxed interaction energy curve is characterized by equivalent minima at *x* = ± 1.7 Å separated by a 1 kcal/mol barrier at *x* = 0 (the sandwich dimer). Points along this curve correspond to the most physically relevant geometries in many chemical and biological systems. Our primary interest is how the introduction of N:, C=O, and NH groups changes the shape of this curve.

**Figure 3:**
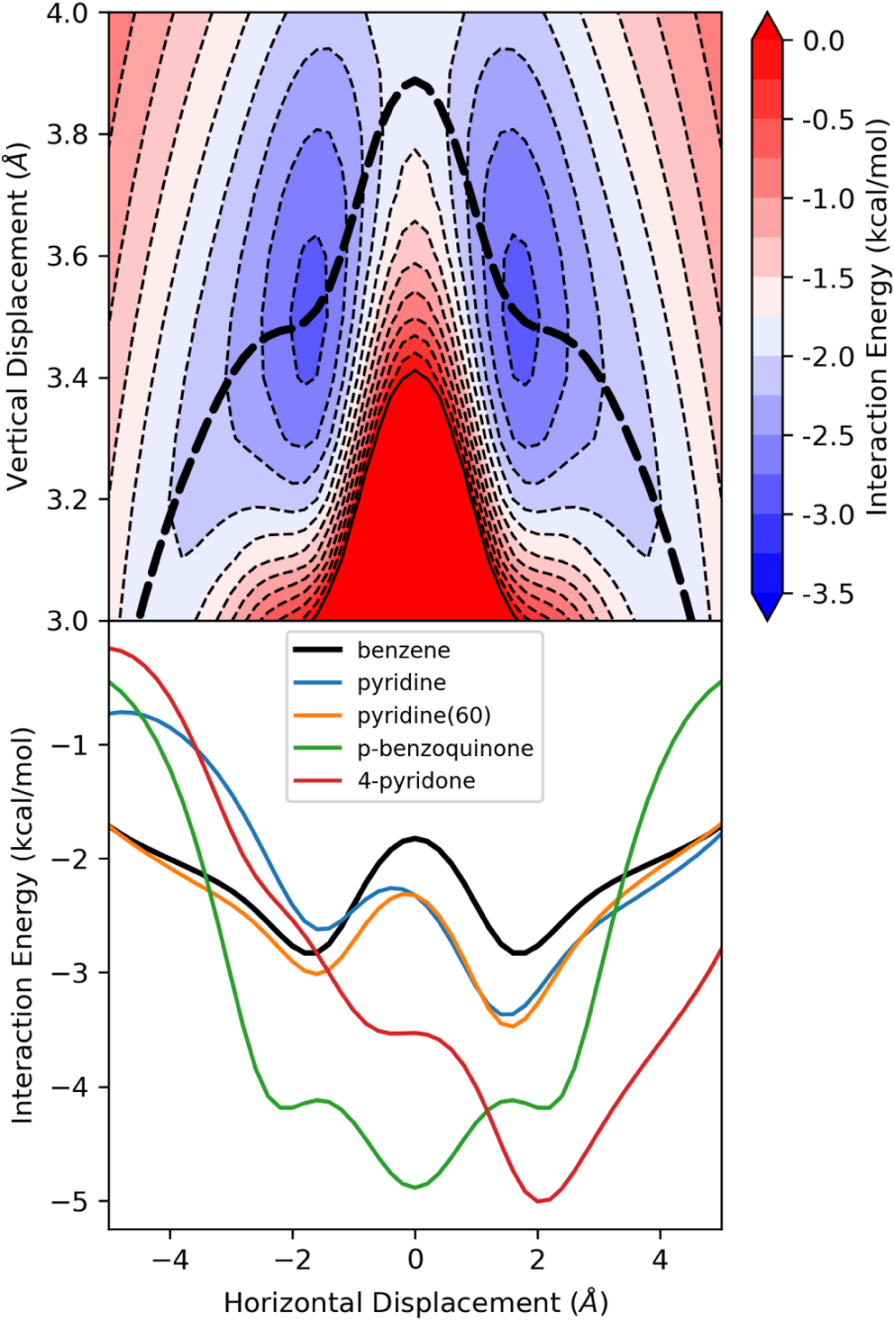
(Top) SAPT interaction energy surface as a function of horizontal and vertical displacements for the parallel stacked benzene dimer. The bold dashed line denotes the minimum energy path (MEP). (bottom) SAPT interaction energies along the corresponding MEPs for the parallel stacked dimers of benzene with selected systems.

The energy associated with the parallel-displaced benzene dimer is considerably wider than that for the benzene-cyclohexane dimer. Zarić et al.^79^ proposed that the spatial extent of the attractive well for parallel stacked aromatic systems is what makes these interactions “special” and ultimately underlies their importance in chemical and biological systems. However, this finding^79^ implicitly assumes that the benzene dimer interaction energy curve is representative of all stacked aromatic systems; as shown below, this does not turn out to be true.

### 3.2 Heteroatom Effects on Parallel Stacking with Benzene

Parallel stacked dimers of benzene with pyridine, *p*-benzoquinone, and 4-pyridone show the range of impacts of heteroatoms on the double-well potential of the benzene dimer (see Figure 3 for relaxed interaction energy curves; see SI Figures S2–S5 for full two-dimensional (2D) interaction potentials). For pyridine, the replacement of a CH group with N induces a tilting and lowering of the interaction energy curve.^22^ The result is an asymmetric double-well potential favoring displacement of the N atom away from the other ring (+*x*) by 0.8 kcal/mol compared to displacement in the opposite direction. Overall, introducing a nitrogen atom enhances stacking for *x* > −1.1 Å and hinders stacking for *x* < −1.1 Å, relative to the benzene dimer. Furthermore, while there is a broad energy well in the +*x* direction, the energy well along the −*x* direction is relatively narrow. In other words, the wide attractive well discussed by Zarić et al.^79^ for the benzene dimer only occurs in one direction for the pyridine-benzene dimer. This has important implications because not only will stacked pyridine-benzene dimers be more favorable if the N atom is displaced away from the other ring, but this interaction will be favorable across a broader range of horizontal distances than for dimers displaced the other direction.

While the nitrogen in pyridine provides a minor perturbation of the benzene dimer potential curve, the introduction of two opposing carbonyl groups leads to a wholesale change in the shape of the potential. That is, for the parallel stacked dimer of benzene with *p*-benzoquinone, there are two shallow minima, corresponding to parallel-displaced geometries, while the global minimum corresponds to the sandwich configuration (*x* = 0)! It should be noted that the elimination of the saddle point at *x* = 0 is not due to changes in the overall topography of the vdW component; the vdW potential for the *p*-benzoquinone-benzene dimer still exhibits the double-well shape characteristic of stacked systems^38,54–56,80^ but is overwhelmed by an electrostatic component that now strongly favors the sandwich configuration (see SI Figure S1).

The overall stacking interaction in the *p*-benzoquinone-benzene dimer is much more favorable than that of the benzene dimer, with the global minimum lying 3 kcal/mol lower in energy than the benzene sandwich dimer and 2 kcal/mol lower than the parallel-displaced benzene dimer. On the other hand, the interaction energy for *p*-benzoquinone stacked with benzene is less favorable for |x| > 3.4 Å. Consequently, the energy well for the parallel stacked dimer of *p*-benzoquinone with benzene is very deep but narrow. Using the criteria from Zarić et al.,^79^ this stacking interaction is not that special!

In the case of 4-pyridone, the introduction of a vinyligous amide has an effect of magnitude similar to that observed for *p*-benzoquinone but with significant asymmetry. The result is the elimination of the minimum along the −*x* direction. Instead, the interaction energy curve features a shelf around *x* = 0 and a single minimum at *x* = 2.1 Å lying more than 2 kcal/mol lower in energy than the benzene dimer minima.

By adding additional N:, C=O, and NH groups and changing their relative positions within the ring, the stacking potential with benzene can be molded to produce a wide range of topographies, well depths, and geometries for parallel-displaced dimers. Doing this purposefully, however, requires an understanding of these heteroatom effects beyond that provided by the Hunter-Sanders Rules.^22,36^ Below, we try to develop that understanding through four key observations about relaxed interaction energy curves for parallel stacked heterocycle-benzene dimers.

#### 3.2.1 Heteroatom Effects Depend Primarily on the Number of Heteroatoms for Sandwich Dimers

For sandwich dimers (*x* = 0) of heterocycles with benzene, the interaction energy is dependent primarily on the number of each heteroatom type; their distribution around the ring is relatively unimportant. For example, the interaction energy of benzene with the three diazines (pyrazine, pyrimidine, and pyridazine) in the sandwich configuration is −2.88 ± 0.03 kcal/mol despite the differences in the relative positions of the N atoms and consequent dipole moments (0.0, 2.4, and 4.2 D, respectively). The situation is the same for the three tetrazines (*E*_int_ = −4.13 ± 0.06 kcal/mol).^81^ Similarly, interaction energies for sandwich dimers of benzene with *o*- and *p*-benzoquinone are nearly identical (−4.87 and −4.89 kcal/mol, respectively). The difference in interaction energy for sandwich dimers of benzene with the two pyridones is slightly larger (−3.36 vs −3.53 kcal/mol for 2- and 4-pyridone, respectively), suggesting a small difference in the impact of amide and vinylogous amide functional groups on stacking in the sandwich configuration.

#### 3.2.2 Heteroatom Effects Depend on the Orientation of the Heteroatom(s) Relative to the Displacement Axis

For parallel stacked dimers with benzene, the shapes of the relaxed interaction energy curves depend primarily on the orientation of each heteroatom relative to the displacement axis (+*x*). For example, Figure 4 shows interaction energy curves for dimers of benzene with the diazines, tetrazines, benzoquinones, and pyridones (2D-interaction energy surfaces are available in SI Figures S6–S13). In general, for each set of isomeric heterocycles, the interaction energy curves differ substantially. However, even though 1,2,3,4-tetrazine has a substantial dipole moment (4.6 D) and 1,2,4,5-tetrazine has zero net dipole, the energy curves for the dimers of these heterocycles with benzene differ by at most 0.1 kcal/mol across all horizontal displacements. The reason for this is that the positions and orientations of each of the four N atoms, relative to the displacement axis, are identical in these two systems. In other words, both 1,2,3,4- and 1,2,4,5-tetrazine have N atoms at angles of 0, 60, 120, and 180° relative to the +*x* axis. Whether the N atoms are spread symmetrically around the ring or clustered on one side has a negligible effect. This can be contrasted with the energy curve for 1,2,3,5-tetrazine, which diverges from those of the other two tetrazines for displaced geometries. The reason is that this tetrazine has two nitrogen atoms at 120° and none at 60°, relative to the +*x* axis.

**Figure 4:**
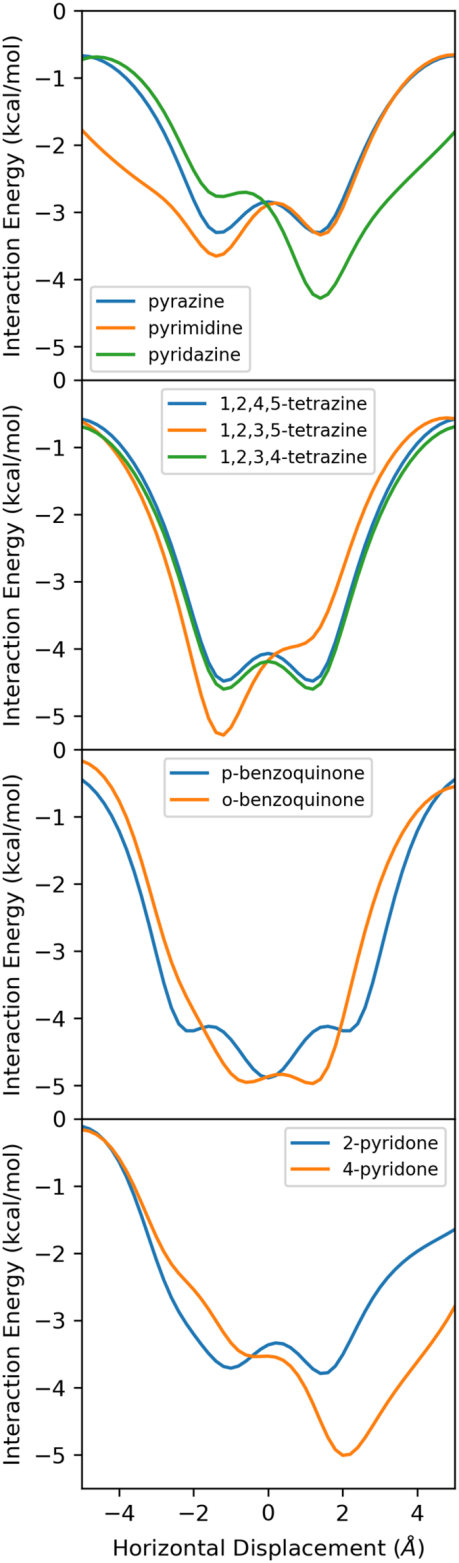
SAPT interaction energy curves for the parallel stacked dimers of benzene with (top to bottom) diazines, tetrazines, benzoquinones, and pyridones.

While the above trend holds consistently across all systems examined, there are also unexpected congruencies across portions of interaction energy curves. Figure 3 shows the relaxed interaction energy curve for pyridine and pyridine(60) stacked with benzene. Intriguingly, for *x* > 0, the two curves closely track each other while the curves diverge for *x* < 0. Instead, the curve for pyridine(60) closely follows that of the benzene dimer for *x* < −2. In other words, for *x* < −2 the N atom in pyridine(60) has a negligible impact on the stacking interaction. Similarly, the interaction energy curves for pyrazine and pyrimidine in Figure 4 differ for *x* < 0, as expected based on the difference in orientation of one of the N atoms relative to the +*x* axis; however, they are indistinguishable for *x* > 0.

#### 3.2.3 Heteroatom Effects Are Additive

The impact of heteroatoms in complex heterocycles stacked with benzene can be captured by considering each heteroatom independently.^82^ For example, the relaxed interaction energy curve for the pyridazine-benzene dimer can be reproduced by adding the energy curves for pyridine and pyridine(60) each stacked with benzene and subtracting the interaction energy curve for the benzene dimer (see Figure 5).^83^ Similarly, the relaxed interaction energy curve for the pyrazine-benzene dimer is reproduced almost quantitatively by adding the energy curves for pyridine and pyridine(180) stacked with benzene and subtracting that of the benzene-benzene dimer (Figure 5).

**Figure 5:**
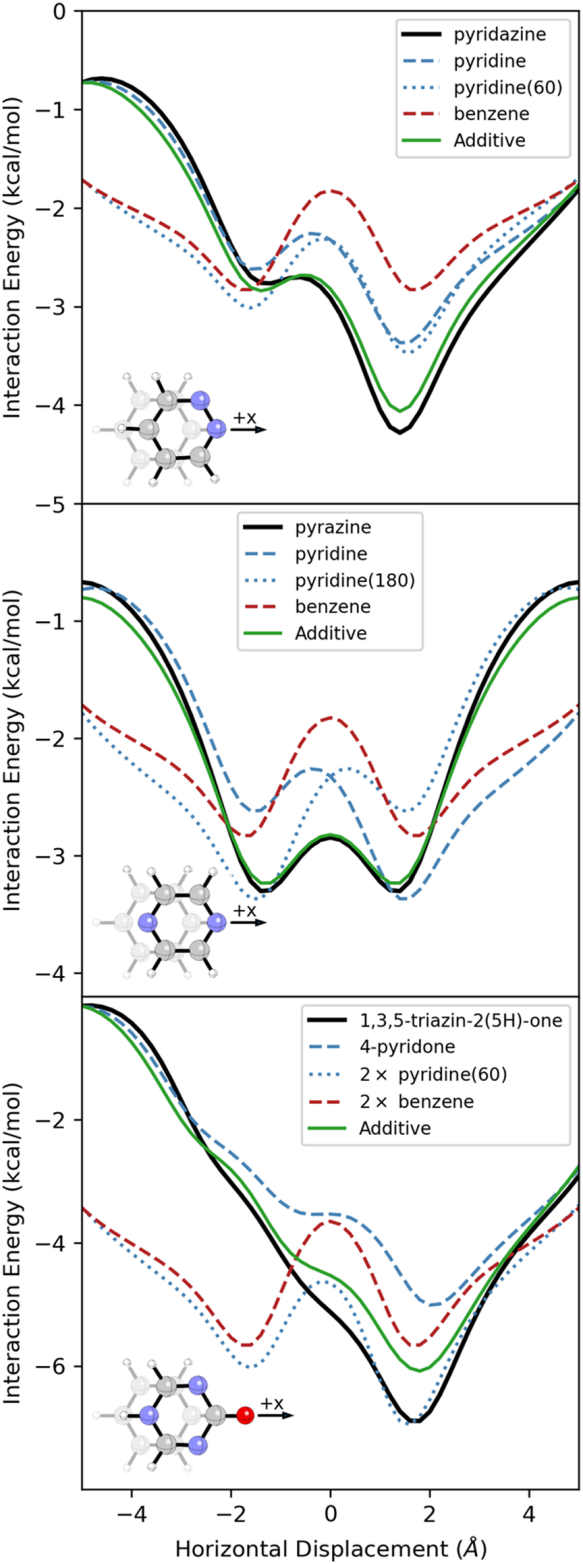
SAPT interaction energy curves for the listed heterocycle with benzene (black), along with the additive approximation (green) arising from the sum of the blue energy curves minus the red energy curve.

This additivity extends to more complex heterocycles and can be exploited to design heterocycle-benzene dimers with the desired interaction energy curves. For instance, while the interaction energy curve for 4-pyridone has a deep energy minimum at *x* = 2.1 Å with a shoulder around *x* = 0, we can add nitrogen atoms at the two α-positions [i.e. 1,3,5-triazin-2­(5*H*)-one] to get an even deeper single-well potential with no shoulder (Figure 5). In this case, the additivity is not exact; for example, the explicitly computed interaction energy at *x* = 1.8 Å is 0.8 kcal/mol more favorable than predicted by this additive model. This appears to be due to the cumulative effects of additional heteroatoms on the MEPs—as more heteroatoms are added, the vertical separation at most horizontal displacements decreases, resulting in a stronger interaction due to charge penetration effects (as seen for polysubstituted benzene dimers).^43,47^ However, qualitatively, we can understand the shape of the interaction energy curve for 1,3,5-triazine-2­(5*H*)-one as the energy curve for 4-pyridone that is further stabilized for +*x* displacements by the pair of N atoms oriented 60° from the +*x* axis.^84^

#### 3.2.4 Heteroatom Effects Are Driven Primarily by Electrostatics

Finally, the stark differences among the relaxed interaction energy curves for heterocycle-benzene dimers are driven primarily by differences in the electrostatic component. Figure 6 shows interaction energy curves for representative heterocycle-benzene dimers compared to curves arising from adding the difference in the electrostatic component between the heterocycle-benzene dimer and the benzene-benzene dimer (along the corresponding MEPs) to the benzene-benzene dimer interaction energy curve:

1 $$\Delta E_{\mathrm{int}}^{\mathrm{het}}≅\Delta E_{\mathrm{int}}^{\mathrm{benzene}}+\left(\Delta E_{\mathrm{elec}}^{\mathrm{het}}-\Delta E_{\mathrm{elec}}^{\mathrm{benzene}}\right)$$

**Figure 6:**
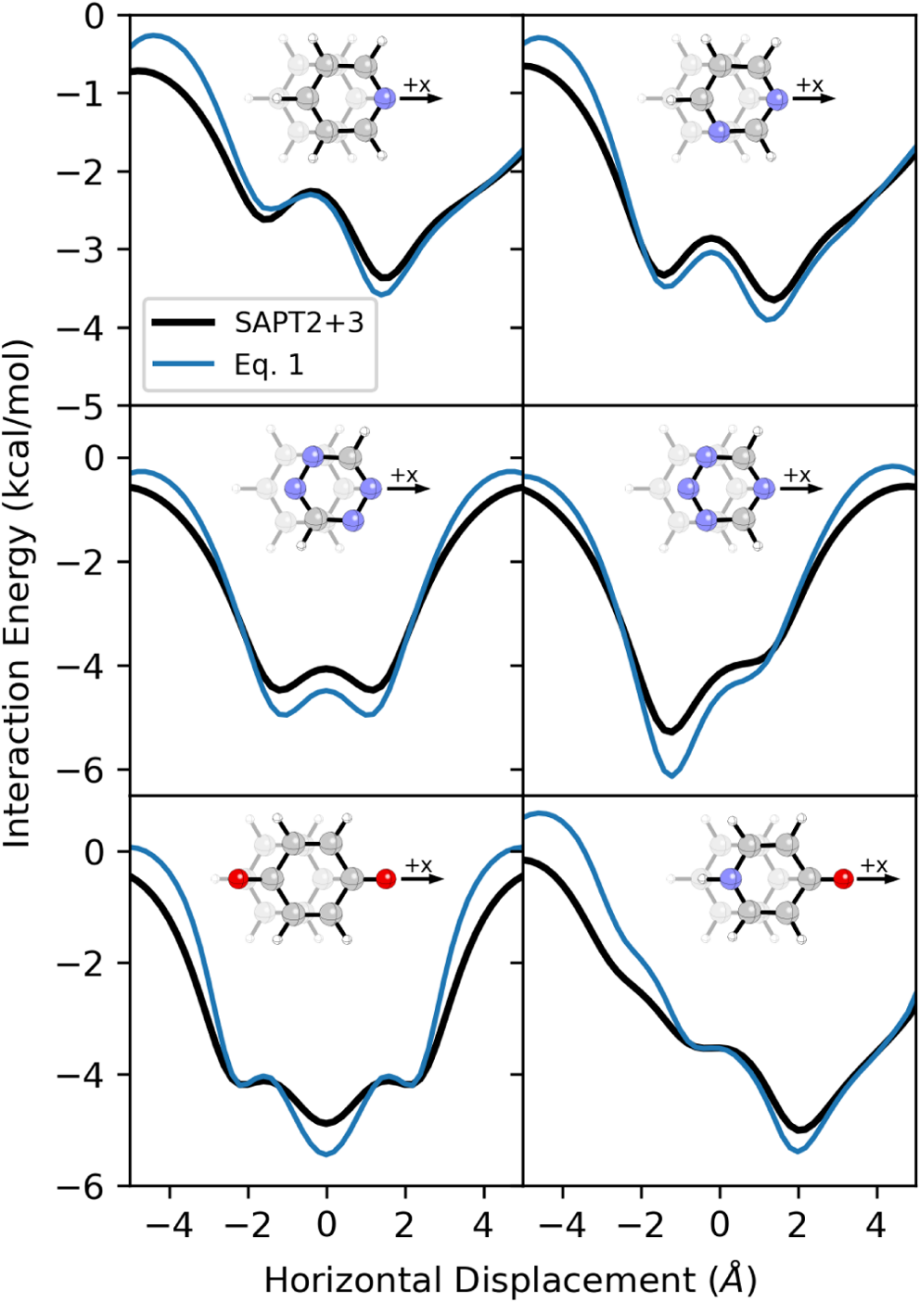
SAPT interaction energy curves (black) for dimers of benzene with selected heterocycles, as well as approximate energy curves (blue) from eq 1.

In these systems (and all systems examined; see SI Figure S17), the resulting approximate interaction energy curve follows the explicitly computed one. To be clear, the interaction energy curves are not quantitatively reproduced by eq 1 and there are significant deviations at certain horizontal displacements due to nonelectrostatic effects. However, the shapes of the energy curves, including the locations and relative energies of the stationary points, are reliably captured. This is not because the differences in the nonelectrostatic components of these interactions are constant along the MEP; they vary wildly (see SI Figure S18). However, these variations are strongly anticorrelated, such that the sum of nonelectrostatic contributions is relatively constant across most horizontal displacements. In other words, as previously observed for stacked dimers at equilibrium geometries,^9,85–89^ the nonelectrostatic components of the interaction energy tend to be balanced along the MEP for stacked dimers of diverse heterocycles with benzene. This balancing of nonelectrostatic effects will be less complete in other regions of the 2D-interaction energy surface, where differences in the vdW component can be significant.^38^ However, if we want to understand how heteroatoms modulate relaxed interaction energy curves, then we can focus solely on the electrostatic component.

### 3.3 A Heuristic Model

To build a conceptual understanding of the heteroatom effects discussed above, we first consider pyridine. Wheeler and Bloom^90^ showed that the impact of replacing CH with N: on both anion-π interactions and the ESPs of azines can be modeled by a point dipole at the position of each N atom. These local radial dipoles have the positive end directed toward the ring centroid (see Figures 7 and S19) and capture the impact of the redistribution of charge within the molecular plane going from C–H (which will have a small local dipole with the positive end toward H) to N:, which will have a more substantial local dipole with the positive end toward the ring centroid. The idea that heteroatom effects can be captured by a dipole-based model was recently exploited by Bootsma and Wheeler^10^ to predict the maximum strength of stacking interactions between drug-like heterocycles and aromatic amino acid side chains^9^ directly from heterocycle connectivity (e.g., SMILES) and is in accord with work from Price and Stone^35^ and Fowler and Buckingham.^34^

**Figure 7:**
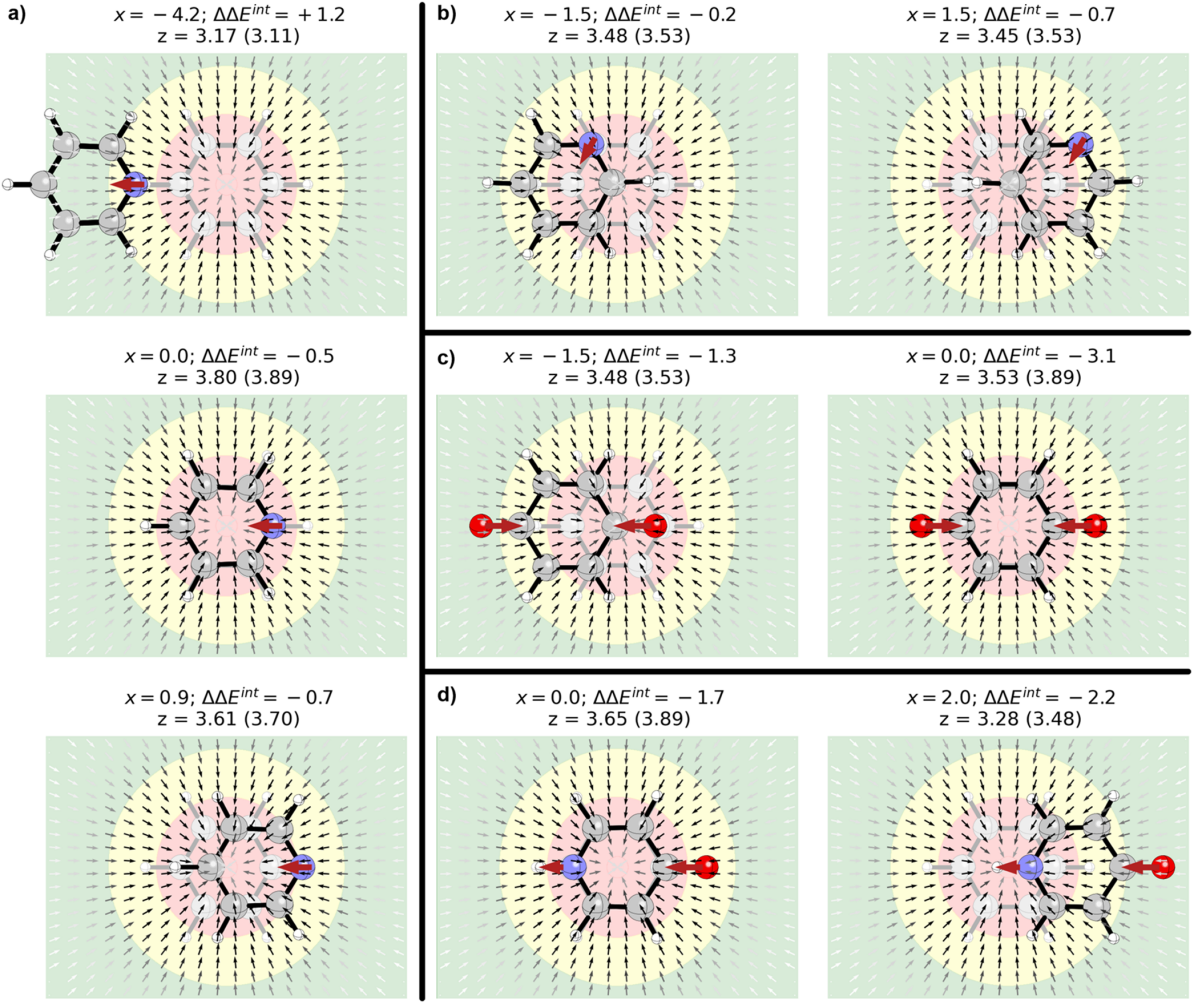
Parallel stacked dimer of benzene with (a) pyridine, (b) pyridine(60), (c) *p*-benzoquinone, and (d) 4-pyridone at selected horizontal displacements (*x*, in Å) along with the ESP and electric field of benzene (darker arrows denote a stronger field). Interaction energies (ΔΔ*E* ^int^), relative to the benzene dimer at the same *x*-value, are given in kcal/mol. Optimized *z*-values for the heterocycle-benzene dimer given in Å (value for the benzene dimer at the corresponding horizontal displacement in parentheses). Red arrows denote the local dipoles representing each heteroatom/functional group.

Applying this to the parallel stacked pyridine-benzene dimer, the interaction energy at a given horizontal displacement can be *approximated* as the interaction of the benzene-benzene dimer at the same displacement plus the interaction of a point dipole at the position of the N atom with the electric field of benzene at that point. The electric field of benzene, 3.5 Å from the molecular plane, is plotted in Figure 7 (see also SI Figure S20). For any parallel plane more than 2 Å from benzene, the electric field points uniformly toward the ring centroid (the electric field above the ring centroid is zero by symmetry). At a typical stacking distance of 3.5 Å, the strength of the field rapidly increases moving away from the centroid before reaching a maximum roughly above the H atoms and then decaying exponentially going away from the ring.

For the pyridine-benzene dimer, in geometries in which the local dipole representing the N atom is aligned with the electric field of benzene, it will have a stabilizing effect. For geometries in which it is antialigned, it will be destabilizing. For example, the pyridine-benzene dimer is depicted in Figure 7a at three horizontal displacements. For *x* = 0 and 0.9 Å, the *N*-dipole is aligned with the electric field, so its presence enhances the stacking interaction; this stabilization is greater for *x* = 0.9 Å because the N atom is positioned over the benzene H atoms, where the electric field is strongest. For *x* = −4.2 Å, the N-dipole is antialigned with the electric field at the point of maximum strength, so the nitrogen is maximally inhibitive of stacking.^91^ Clearly, this simple dipole model is not quantitatively correct. For instance, there is an asymmetry in the impact of the N atom of pyridine (the destabilizing effect at *x* = −4.1 Å is greater than the stabilizing effect at *x* = 0.9 Å), not accounted for by this simple model.^92^ Regardless, it captures the main differences between the pyridine-benzene and benzene-benzene interaction energy curves.

For heteroatoms not aligned with the displacement axis, we need to account for the angle of the corresponding dipole relative to the electric field at that position (the interaction of a dipole with an electric field depends on the dot product of the corresponding vectors). Figure 7b shows two geometries of the pyridine(60)-benzene dimer. For *x* = −1.5 Å and larger displacements, the N-dipole is nearly perpendicular to the benzene electric field, so there is minimal impact on the interaction energy for these geometries (see Figure 3). On the other hand, for + *x* displacements, the N-dipole remains roughly parallel to the electric field, so it enhances the interaction relative to the benzene dimer. While the dipole-field alignment is not exactly parallel for the N atom in pyridine(60), like it is for the N-dipole in pyridine, the nitrogen in pyridine(60) is closer to the benzene centroid than the nitrogen in pyridine for all +*x* displacements (see SI Figure S28). Consequently, for a given horizontal displacement, the N-dipole in pyridine(60) interacts with a slightly stronger electric field than the N atom in pyridine. The effect of this stronger field seems to balance the diminished heteroatom effect due to the suboptimal dipole-field alignment, resulting in interaction energy curves for pyridine and pyridine(60) that are very similar for *x* > 0 (see Figure 3).

While this dipole-field picture works best for N atoms,^93^ the impact of C=O and NH groups can be approximately captured by considering local dipoles of the right magnitude and direction (see Figure 7c,d). For example, the global minimum energy geometry for the dimer of benzene with *p*-benzoquinone corresponds to the sandwich configuration (see Figure 3). This geometry places both carbonyl dipoles parallel to the electric field near the point of maximum strength (see Figure 7c), leading to more than 3 kcal/mol enhancement in the stacking interaction relative to the benzene sandwich dimer. Any horizontal displacement results in the reduction of both dipole-field interactions and a subsequent reduction in stacking strength. For displacements beyond |*x*| = 3.4 Å, one carbonyl dipole is aligned with a greatly diminished electric field, while the other is antialigned with the field near its maximum. The result is that the overall stacking interaction in the *p*-benzoquinone-benzene dimer is weaker than that of the benzene dimer for these large lateral displacements.

Similarly, the sandwich dimer of benzene with 4-pyridone stacks 1.7 kcal/mol more strongly than the benzene-benzene dimer in the same geometry. In this case, the carbonyl dipole is aligned with the electric field at the corresponding position while the NH-dipole is antialigned (see Figure 7d); the former interaction will be stabilizing while the latter will be destabilizing. However, the local CO-dipole is assumed to be larger than the NH dipole^10^ and is located closer to the point of maximum electric field. The result is a net stabilization of the 4-pyridone-benzene dimer. For *x* = 2.0 Å, the stabilizing interaction between the CO-dipole and the benzene electric field is mostly retained, while the destabilizing interaction of the field with the NH-dipole is gone because the NH is now positioned over the benzene centroid. This result is an even greater (2.2 kcal/mol) enhancement of stacking.

We want to be abundantly clear that this is a qualitative model that relies heavily on the cancellation of errors. For example, we are comparing interaction energies of dimers at a given horizontal displacement but at different corresponding optimal vertical separations. For carbonyl-containing heterocycles, in particular, the vertical separations are often substantially different from those of benzene at a given lateral displacement. Similarly, we are visualizing the electric field of benzene in a single plane (*z* = 3.5 Å), yet the heteroatoms will be at different vertical positions depending on both the nature of the heterocycle and the horizontal displacement. The point here is not to develop a rigorous quantitative model of heteroatom effects; the goal is an intuitive qualitative model that provides reliable predictions without computations.

### 3.4 T-Shaped Dimers

So far, we have focused on parallel stacking interactions; however, aromatic systems often interact in perpendicular orientations.^94–105^ The strength of these T-shaped interactions can also be tuned through the introduction of heteroatoms. Figure 8 shows the effect of introducing a nitrogen atom at different positions in one ring of the T-shaped benzene dimer on the interaction energies along the corresponding MEPs (2D potentials are available in SI Figures S22–S24). While these heteroatom effects do not change the overall shape of the potential energy curve, they do change the depth and symmetry of the energy well. Once again, these heteroatom effects are additive. For instance, the relaxed interaction energy curve for the T-shaped pyrimidine-benzene dimer can be captured by summing the energy curves for the corresponding pyridine-benzene dimers and subtracting the curve for the T-shaped benzene dimer (see SI Figure S25). Furthermore, as seen for the parallel stacked dimers, the differences among these energy curves are dominated by electrostatic effects (SI Figure S26), meaning that we can understand them in terms of local dipoles and electric fields.

**Figure 8:**
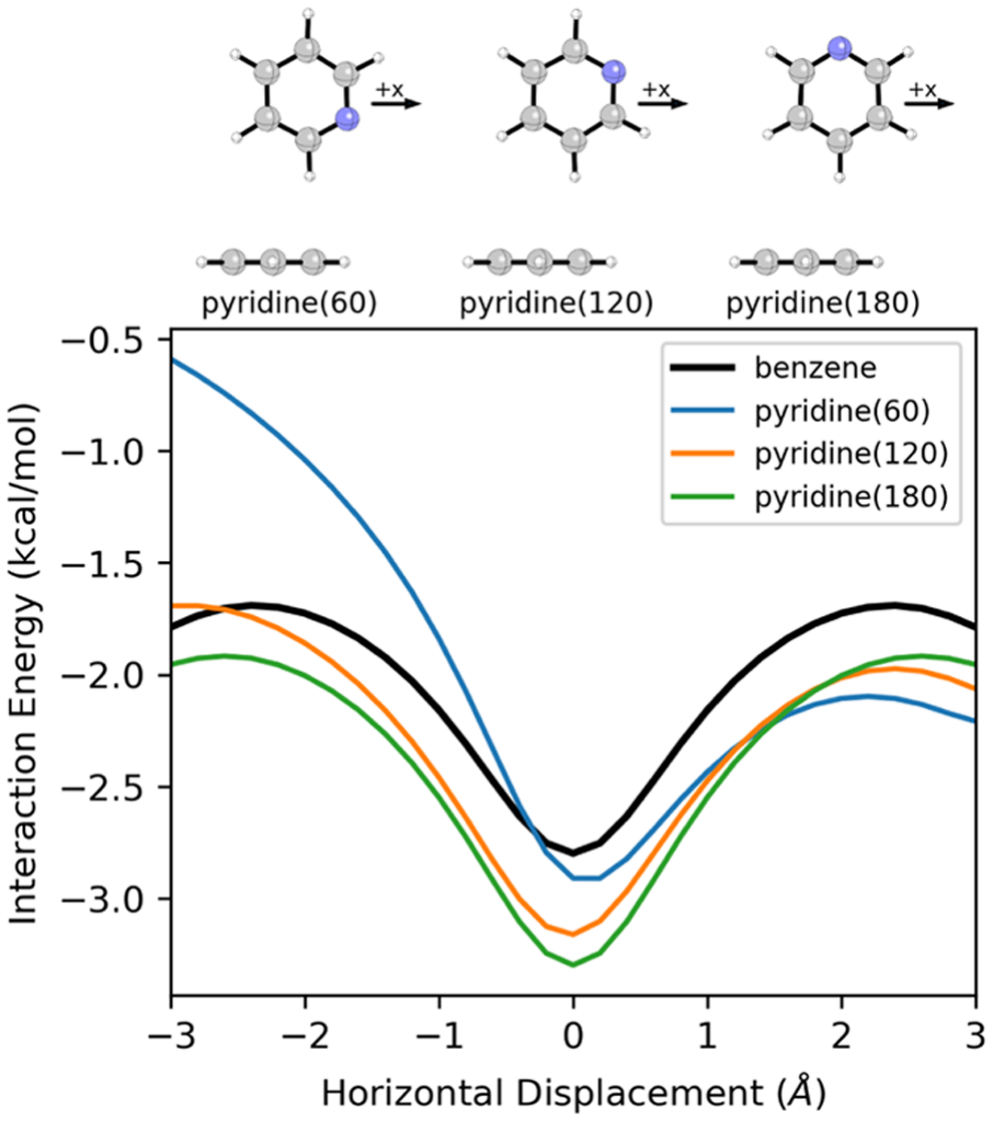
SAPT interaction energy curves for T-shaped dimers of benzene with benzene and pyridine in three orientations.

For the T-shaped pyridine(60)-benzene dimer, the interaction is enhanced for +*x* displacements and hindered for −*x* displacements, relative to the T-shaped benzene-benzene dimer (see Figure 8). For +*x* displacements, the N-dipole is partially aligned with a relatively strong electric field (see Figure 9), whereas it is antialigned for −*x* displacements. This can be contrasted with the T-shaped dimers of pyridine(120) and pyridine(180) with benzene. In these systems, the nitrogen atom enhances the interaction across all horizontal displacements because the N-dipole is always at least partially aligned with the electric field of benzene. The alignment is slightly worse at most geometries for pyridine(120) than for pyridine (180), but this is partially offset by the N-dipole interacting with a slightly stronger electric field in the former case. The slight asymmetry of the interaction energy curve for pyridine(120) (+*x* more favorable than −*x* displacements) can also be explained by the more optimal alignment of the dipole with the electric field for +*x* displacements.

**Figure 9:**
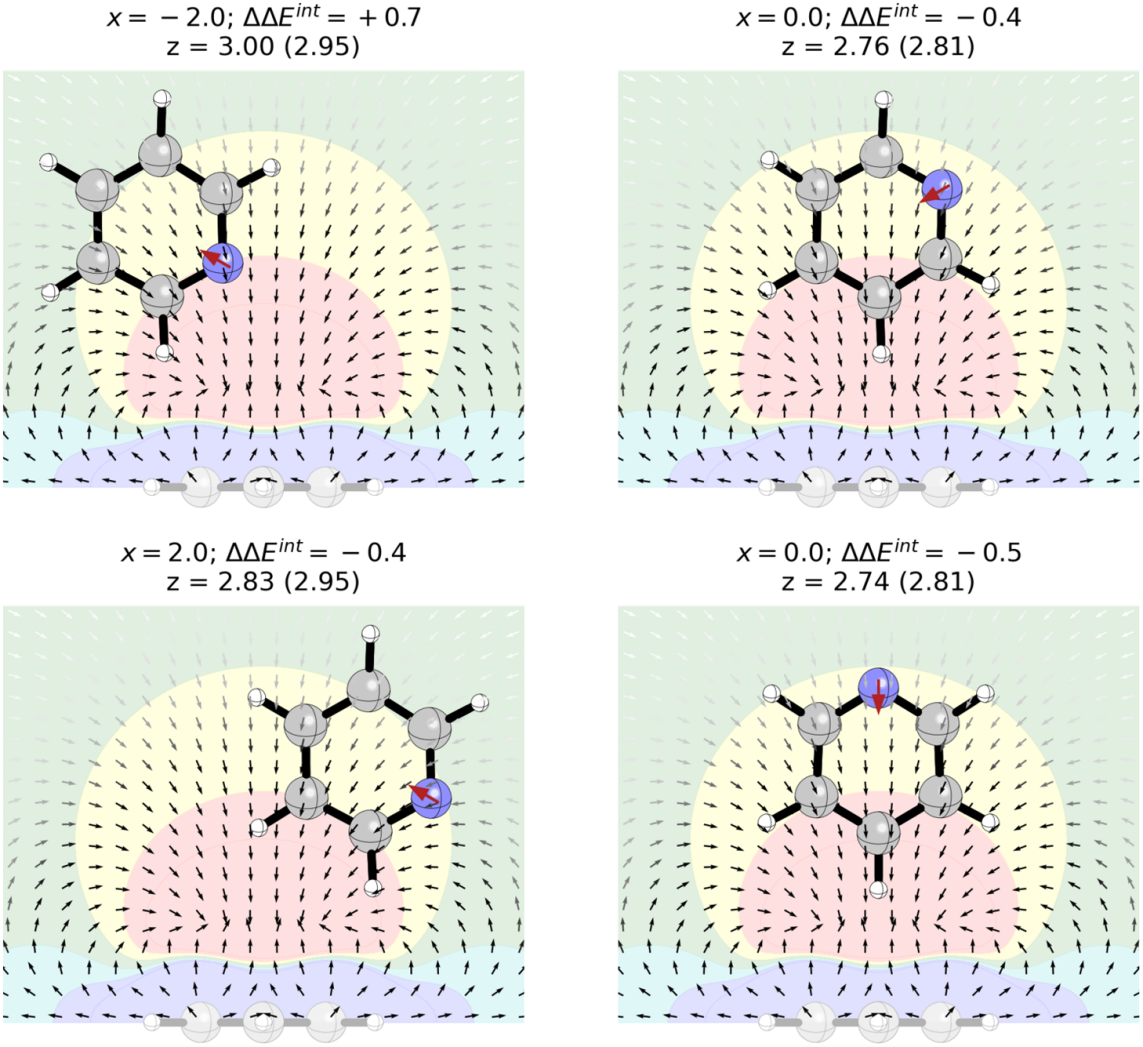
T-shaped dimers of benzene with pyridine in different orientations at selected horizontal displacements (*x*, in Å) along with the ESP and electric field of benzene. Interaction energies (ΔΔ*E* ^int^), relative to the T-shaped benzene dimer at the same *x*-value, are given in kcal/mol. Optimized distance between the bottom ring and the closest H of the top ring for the heterocycle-benzene dimers (*z*) is given in Å (value for the benzene dimer in parentheses). Red arrows denote the local dipole representing the nitrogen atom.

### 3.5 More Complex Dimers

In the previous sections, we considered how the introduction of heteroatoms into one ring can change the interaction energy curve of the benzene dimer. However, there is nothing unique about the benzene dimer as a reference! We can understand heteroatom effects in more complex heterocyclic dimers relative to the appropriate reference dimer in terms of the interaction of local dipoles associated with the heteroatoms in one system with the electric field of the other.

Some of the key results from Section 3.2 for heterocycle-benzene dimers need to be modified slightly when considering stacked dimers involving less-symmetric arenes. For instance, in sandwich dimers involving two heterocycles, the relative positions of heteroatoms on opposing systems will often be critical because for most heterocycles the electric field is of lower symmetry than that of benzene. Similarly, the effect of heteroatoms in one ring will not be strictly determined by their orientation relative to the displacement axis unless the electric field of the other system is symmetric across this axis. However, other key results from Section 3.2 apply to all parallel stacked heterocyclic dimers.

For example, Figure 10 shows the relaxed interaction energy curve for pyrazine(120) stacked with 2-pyridone. The introduction of the two nitrogen atoms in pyrazine significantly enhances the stacking interaction, particularly for *x* > 0. The net result is that while the interaction energy curve for 2-pyridone stacked with benzene has two roughly equal minima, the parallel stacked dimer of 2-pyridone with pyrazine(120) strongly favors +*x* displacements. As seen for the heterocycle-benzene dimers, the effects of the two N atoms in pyrazine are additive (see Figure 10). That is, the energy curve for pyrazine(120) with 2-pyridone is reproduced by adding the corresponding energy curves for pyridine(120) and pyridine(−60) stacked with 2-pyridone and subtracting the interaction energy curve for the dimer of benzene with 2-pyridone.

**Figure 10:**
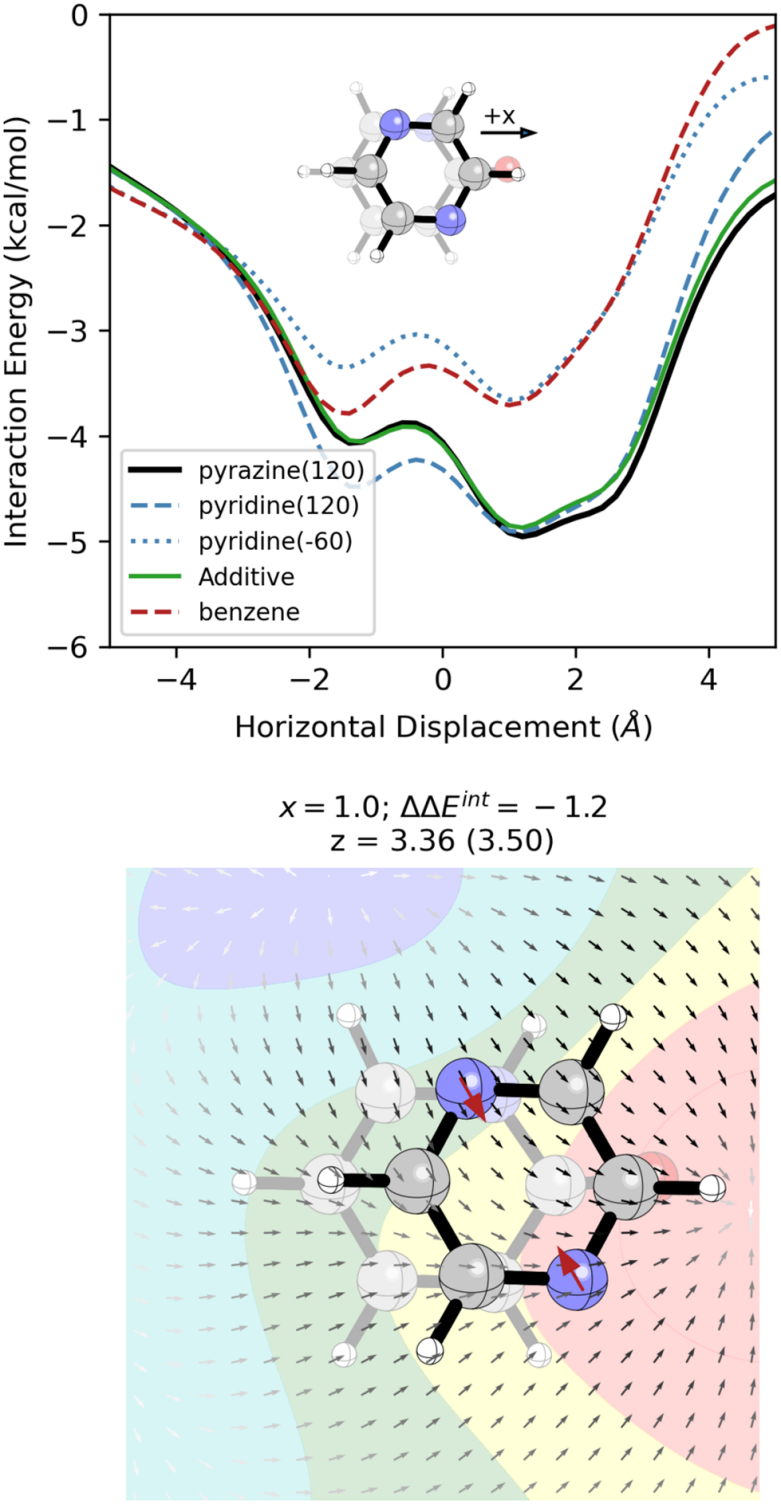
(Top). SAPT interaction energy curve for pyrazine(120) stacked with 2-pyridone (black), along with additive approximation (green) arising from the sum of the blue energy curves minus the red energy curve. (bottom) Stacked dimer of pyrazine(120) with 2-pyridone at *x* = 1.0 Å, along with the ESP and electric field of 2-pyridone in the *z* = 3.5 Å plane. The interaction energy (ΔΔ*E* ^int^), relative to the 2-pyridone-benzene dimer at the same *x*-value, is given in kcal/mol. Optimized z-value for the pyrazine(120)-2-pyridone dimer is given in Å (value for the benzene-2-pyridone dimer is in parentheses). Red arrows denote the local dipoles representing the nitrogen atom.

The changes in the interaction energy curve due to the two N atoms are also dominated by electrostatic effects (see SI Figure S27) and can be explained in terms of the interaction of the two N-dipoles with the electric field of 2-pyridone. As shown in Figure 10, the N atom from pyridine(120) is aligned with the electric field of 2-pyridone across all horizontal displacements; the field experienced by this N is strongest for *x* > 0, leading to a more substantial enhancement of stacking across these horizontal displacements. The N-dipole from pyridine(−60), on the other hand, is orthogonal to the electric field of 2-pyridone for *x* > 0 and antialigned with a much weaker field for *x* < 0. This explains why the pyridine(−60) nitrogen has little impact for +*x* displacements and hinders stacking for −2 < *x* < 0.

Overall, viewing heteroatom effects through the lens of local dipoles and electric fields provides a powerful means of understanding complex stacked dimers. While we have focused on relaxed potential energy curves for horizontal displacements along one axis for six-membered rings, there is nothing unique about this axis or monocyclic systems. The above model will apply to heterocyclic dimers for any lateral displacement or orientation if the vertical distance is optimal. As an example, we consider the global minimum energy parallel stacked dimers of benzene and pyridine with the nucleobase guanine.^89^ Understanding how to modulate the strength of such interactions is vital for the rational design of RNA-binding small molecules.^106–116^ As previously observed by Conner et al.,^89^ the introduction of a nitrogen minimally perturbs the preferred geometry of the benzene-guanine dimer yet results in a 2.2 kcal/mol enhancement in interaction energy (see Figure 11). The electric field of guanine shows that in this orientation, the dipole associated with the N atom of pyridine is perfectly aligned with the electric field of guanine at that position; any other orientation of pyridine would result in the local dipole either antialigned or perpendicular to the field. Looking at this another way, Figure 11 shows that replacement of any additional CH group with N: in this binding pose, will, at best, leave the stacking interaction unchanged.

**Figure 11:**
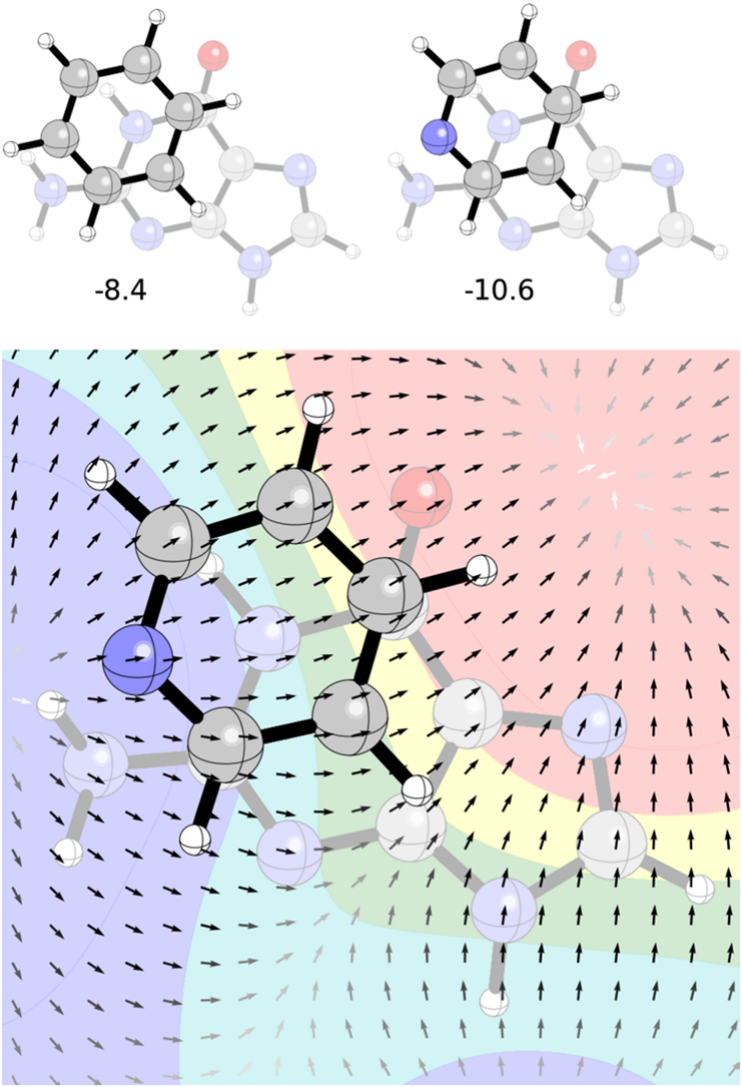
(Top) Global minimum energy dimers of benzene and pyridine with guanine from Conner et al.^89^ [DLPNO–CCSD­(T) interaction energies in kcal/mol]; (bottom) ESP and the electric field of guanine in the plane 3.5 Å away.

This example provides key insight into a vexing problem in drug design: where to place nitrogen atoms to maximally enhance stacking interactions with binding site aromatic groups. The dipole-field model presented above suggests that, in general, stacking will be enhanced by replacing any CH group(s) whose H–C bond vector(s) are aligned with the electric field of the other aromatic system. A quantitative implementation of this concept is described in a separate paper.

## 4 Concluding Remarks

Heteroatoms provide a powerful means of controlling the strength of parallel and T-shaped interactions between aromatic systems. Directly predicting the interaction energy for a given heteroaromatic dimer requires robust quantum-mechanical computations.^38,60^ However, for many applications, all that is required is the interaction energy of a given dimer relative to some reference dimer in a similar geometry. In other words, we often just need to know how heteroatoms will *change* the strength of a given interaction. This change is much easier to predict.

Above, we showed that N:, C=O, and NH groups can change the number and location of energy minima along the relaxed interaction energy curve as a function of horizontal displacements for the parallel stacked benzene dimer. These changes are additive, dependent primarily on the orientation of the heteroatoms relative to the displacement axis, and driven mostly by electrostatic effects. These features justify a heuristic model of heteroatom effects in π-π interactions in which the impact of a given heteroatom is described by the interaction of a local dipole at the position of the heteroatom (with the size and direction dependent on the heteroatom type) with the electric field of the other aromatic system. We showed how this model can be used to understand heteroatom effects in T-shaped interactions as well as dimers of complex heterocycles.

This dipole-field model is qualitative and will obviously not provide robust predictions for all aromatic dimers. For instance, Figure 5 already shows a breakdown in the additivity that is implicit in this model, and Figure 6 shows that nonelectrostatic effects can contribute significantly to the interaction energy at certain horizontal displacements. Clearly, this model will never provide predictions with accuracy rivaling that of modern *ab initio* methods. That is not the goal; the goal is to provide a heuristic model that enables chemists to harness heteroatom effects to design aromatic molecules that exhibit the desired stacking behavior without having to resort to computations.

There are, of course, additional caveats. For instance, we considered only idealized (perfectly parallel or perpendicular) gas-phase dimers. Realistic systems will exhibit more varied geometries, will often occur in trimers, tetramers, etc., and will generally be impacted by solvent or other dielectric environments. Nonetheless, the model presented above should serve as a sound starting point for predicting heteroatom effects on noncovalent interactions between aromatic systems, with other complicating factors accounted for through other means. At the same time, although there are some nonadditivity effects in aromatic clusters,^117–124^ in terms of qualitative predictions, an assumption of pairwise additivity is justifiable. To this end, we note that electric fields are additive, so we should be able to understand heteroatom effects on the central ring of a stacked trimer, for example, in terms of the interaction of local dipoles interacting with the sum of the electric fields of the flanking aromatic groups.

That this dipole-based model works will seem counterintuitive (perhaps even absurd) to many.^125^ One reason is that it ignores the impact of heteroatoms on the distribution of π-electrons, which contrasts with the original Hunter-Sanders model,^36^ the later models from Hunter et al.,^126^ and most chemists’ intuition regarding aromatic systems! But it works. The reason is that the primary means through which heteroatoms change the electrostatic character of an aromatic system is through the redistribution of charge within the molecular plane;^31,34,90^ any changes in the π-electron distribution have a negligible impact on noncovalent interactions. This is in accord with our work on substituent effects in stacking interactions,^45,49,51,127–131^ which themselves can be explained (in part) in terms of a local dipole associated with each substituent interacting with the electric field of the other ring.^131^

Finally, we note that interactions between aromatic systems have long been discussed in terms of ESPs (e.g., “electron-rich” vs “electron-poor”).^1,3,41,43,50,60,126,128,132–136^ The model presented above emphasizes that in terms of heteroatom effects, the sign of the ESP of the opposing aromatic system is immaterial; what is important is the gradient of the ESP (i.e., the electric field) of one aromatic system at the positions of the heteroatoms in the other.

## Supplementary Material









## Notes

SAPT data for parallel stacked dimers parallel_SAPT (XLSX)
SAPT data for T-shaped dimers T-shaped_SAPT (XLSX)
Cartesian coordinates of all monomers (XYZ)
Additional figures and computational details (PDF)

## †

Division of Chemistry and Chemical Engineering, California Institute of Technology, Pasadena, CA 91125, United States
