# Supplementary material for: Making Sense of Heteroatom Effects in π–π Interactions
Source: J Am Chem Soc. 2025 Aug 21;147(35):32273–86. doi: 10.1021/jacs.5c12769 (PMC12412153; doi:10.1021/jacs.5c12769)
Supplement: Supplementary file 4 [file ja5c12769_si_004.pdf]

Supporting Information

Making Sense of Heteroatom Effects in π-π Interactions

Khue U. Do, Audrey V. Conner, and Steven E. Wheeler*

Department of Chemistry, University of Georgia, Athens, GA 30602 United States

E-mail: swheele2@uga.edu

Contents **page**

I. Computational Details and Sample Input Files S2

II. Additional Figures S3

Figure S1. vdW and electrostatic components for parallel stacked dimers of benzene and p-benzoquinone with benzene S5

Figures S2-S16. 2-dimensional interaction energy surfaces for parallel stacked dimers S6

Figure S17. SAPT interaction energies compared to predicitons from Eq. 1 for additional dimers S21

Figure S18. Difference in SAPT energy components for benzene-heterocycle dimers compared to benzene-benzene dimers S22

Figure S19. Additional example of the pyridine-benzene dimer explained in terms of the dipole-field model S23

Figure S20. ESP and electric field of benzene S24

Figure S21. Parallel stacked pyridine-benzene at x = -1.1 along with the ESP and electric field of benzene S25

Figure S22-24. 2-dimensional interaction energy surfaces for T-shaped dimers S26

Figure S25. Addivitity of heteroatom effects in the T-shaped pyrimidine-benzene dimer S29

Figure S26. SAPT interaction energies compared to predicitons from Eq. 1 for T-shaped dimers of pyridine with benzene S30

Figure S27. SAPT interaction energies compared to predicitons from Eq. 1 for the parallel stacked dimer of pyrazine(120) with 2-pyridone S31

Figure S28. Distances between N atom in pyridine and pyridine(60) relative to benzene in parallel stacked pyridine and pyridine(60)-benzene dimers S31

III. References S32

I. Computational Details and Sample Input Files

Interaciton energies were computed at the SAPT2+3/def2-TZVPD level of theory[1-3] using Psi4.[4] In Ref. [5], we showed that this level of theory very closely approximates the relaxed interaction energy curve for the parallel stacked benzene dimer at the SAPT2+3(δMP2)/aug-cc-pVTZ level of theory, which is generally considered the 'gold standard' among SAPT methods,[6] but at a reduced computational cost. Monomers were frozen at their wB97X-D/def2-TZVP optimized geometries (computed using Gaussian16).[7] These monomers were then aligned with the benzene dimer to ensure that horizontal displacments of $x = 0$ correspond to aligned ring centroids. For parallel stacked dimers, two-dimensional scans over horizontal (x) and vertical (z) displacements of the 'top' ring for $x = -5$ to +5 Å in 0.2 Å increments and $z = 2.6$ to 4.0 Å in 0.1 Å increments were done using AaronTolols.[8] For the T-shaped dimers, we scanned from $x = -3$ to +3 Å in 0.2 Å increments and $z = 4.5$ to 6.0 Å in 0.1 Å increments. In both cases, for each x-value, the total interaction energy was fit to a cubic spline interpolator and the minimum energy point along this interpolated curve found to identify the minimum energy path (MEP) across this 2-dimensional surface. The components of the SAPT interaction energy were also fit to cubic spline interpolators and evaluated at the z-values of the MEP. Molecular structure images in Figures 5-11 and the SI Figures generated using AaronTools.[8]

Gaussian input file for monomer optimization (for benzene):

```
#n wb97xd/def2tzvp Opt sym=loose

Benzene (D6h)

0 1
C       0.95157     1.00936    -0.00000
C       1.34995    -0.31940    -0.00000
C       0.39838    -1.32879     0.00000
C      -0.95157    -1.00936     0.00000
C      -1.34995     0.31940     0.00000
C      -0.39838     1.32879    -0.00000
H       1.69431     1.79731    -0.00000
H       2.40368    -0.56874    -0.00000
H       0.70934    -2.36601     0.00000
H      -1.69431    -1.79732     0.00000
H      -2.40368     0.56875     0.00000
H      -0.70934     2.36601    -0.00000
```

Psi4 input file for SAPT2+3 computation for benzene dimer at (x, z) = (3.0, 3.8):

```
molecule dimer {
0 1
C     -1.38719    0.00000    0.00000
C     -0.69359   -1.20134    0.00000
C      0.69359   -1.20134    0.00000
C      1.38719   -0.00000    0.00000
C      0.69359    1.20134    0.00000
C     -0.69359    1.20134    0.00000
H     -2.47002    0.00000    0.00000
H     -1.23501   -2.13910    0.00000
H      1.23501   -2.13910    0.00000
H      2.47002   -0.00000    0.00000
H      1.23501    2.13910    0.00000
H     -1.23501    2.13910    0.00000
--
0 1
C      1.61281    0.00000    3.80000
C      2.30641   -1.20134    3.80000
C      3.69359   -1.20134    3.80000
C      4.38719    0.00000    3.80000
C      3.69359    1.20134    3.80000
C      2.30641    1.20134    3.80000
H      0.52998    0.00000    3.80000
H      1.76499   -2.13910    3.80000
H      4.23501   -2.13910    3.80000
H      5.47002    0.00000    3.80000
H      4.23501    2.13910    3.80000
H      1.76499    2.13910    3.80000
units angstrom
}
set globals {
  basis def2-tzvpd
  scf_type DF
  guess sad
  freeze_core True
}
memory 24 Gb
energy('sapt2+3')
```

Python program for plotting electric field and ESP calculated using Psi4 (reproducing top of Figure S20):

```
# Plot ESP and E-field computed using Psi4
# requires: AaronTools, Psi4, matplotlib, numpy
from AaronTools.geometry import Geometry
import psi4
import numpy as np
import matplotlib.pyplot as plt

fig, ax = plt.subplots()

# read geometry for benzene (located in xy-plane)
geom = Geometry('benzene.xyz')

# Convert to Psi4 molecule
mol = geom.convert_to_Psi4()

z = 3.5 # z-value for plane in which ESP and field are computed
fmax = 0.5   # maximum for field scale
fmin = 0.024 # minimum field to plot
Xmin = -4
Xmax = 4
Ymin = -4
Ymax = 4
alpha = 0.5

#Make meshgrid for contour plot
xgrid = np.linspace(Xmin, Xmax, 100)
ygrid = np.linspace(Ymin, Ymax, 100)
x, y = np.meshgrid(xgrid, ygrid)

# compute ESP using Psi4
points = np.vstack([x.ravel(), y.ravel()] + [np.array([z]*xgrid.size*ygrid.size)]).T
psi4_matrix = psi4.core.Matrix.from_array(points)

# get wavefunction
psi4.set_output_file("output.dat", False)
energy, wfn = psi4.energy('wb97x-d/def2-tzvp', return_wfn = True)
myepc = psi4.core.ESPPropCalc(wfn)

# Compute ESP on grid
psi4.set_num_threads(8, quiet = True)
ESP = 627.51*np.array(myepc.compute_esp_over_grid_in_memory(psi4_matrix))

# Convert ESP to 2D array and make contour plot
ESP = ESP.reshape(x.shape)
ax.set_aspect('equal')
scale=np.arange(-7.5, 7.6, 3)

# coordinates
xs = (points[:,0]).reshape(x.shape)
ys = (points[:,1]).reshape(y.shape)

CSF = ax.contourf(xs, ys, ESP, scale,
colors=('r','yellow','g','c','b'),alpha=alpha,vmax=abs(ESP).max(), vmin=-
abs(ESP).max(),extend='both', zorder=-10)
CSF.cmap.set_under('red')
CSF.cmap.set_over('blue')

# Plot E field arrows (colored by magnitude)
# cut edges off to avoid arrows that extend beyond the plot
xgrid = np.linspace(Xmin + 0.1, Xmax - 0.1, 20)
ygrid = np.linspace(Ymin + 0.1, Ymax - 0.1, 20)
x, y = np.meshgrid(xgrid, ygrid)
points = np.vstack([x.ravel(), y.ravel()] + [np.array([z]*xgrid.size*ygrid.size)]).T
psi4_matrix = psi4.core.Matrix.from_array(points)

# coordinates
xs = (points[:,0]).reshape(x.shape)
ys = (points[:,1]).reshape(y.shape)
```

```
# calculate E-field
Field_data = np.array(myepc.compute_field_over_grid_in_memory(psi4_matrix))

# Convert to kcal/mol/D
F_x=Field_data[:,0]*627.51/2.542
F_y=Field_data[:,1]*627.51/2.542

# Normalize E-field vectors
F_strength=np.sqrt(F_x*F_x + F_y*F_y)
F_x /= F_strength
F_y /= F_strength

# Apply fmax cutoff
F_strength[F_strength > fmax] = fmax

# Zero out any field vectors below fmin
weak_field_idx = F_strength < fmin
F_x[weak_field_idx] = 0
F_y[weak_field_idx] = 0
F_strength[weak_field_idx] = fmin

# Plot Field arrows
ax.quiver(xs, ys, F_x, F_y, F_strength, alpha=1, cmap=plt.cm.Greys, zorder=5)

# Plot AaronTools geometry
geom.plot(ax, fig)

plt.savefig("example.tif", transparent=True, dpi=300, bbox_inches='tight')
```

II. Additional Figures

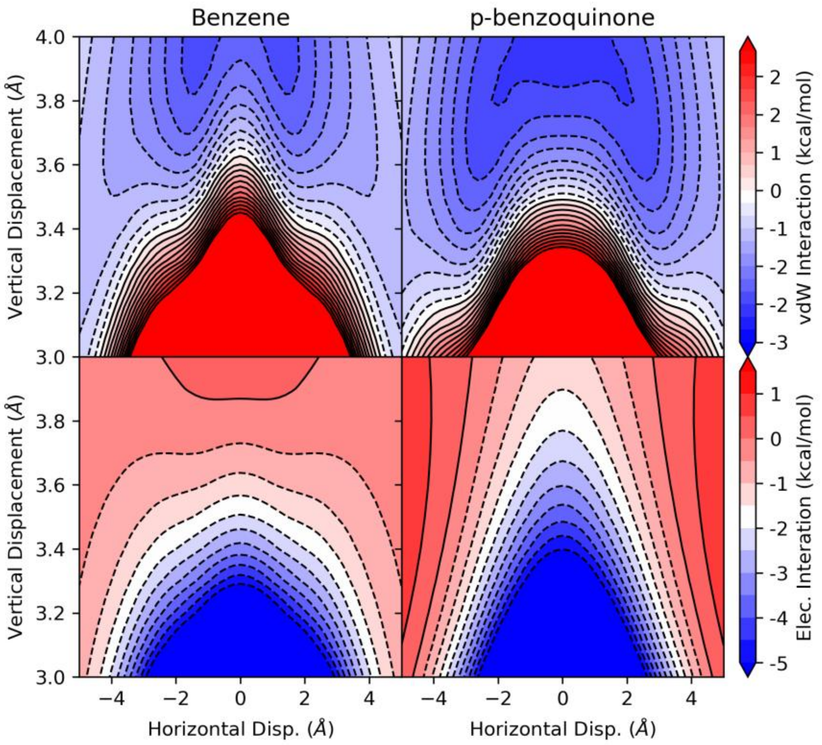

Figure S1. vdW (top) and electrostatic (bottom) componoents of the interaction energy for the parallel stacked dimers of benzene with benzene (left) and p-benzoquinone (right) as a function of horizontall and vertical displacements.

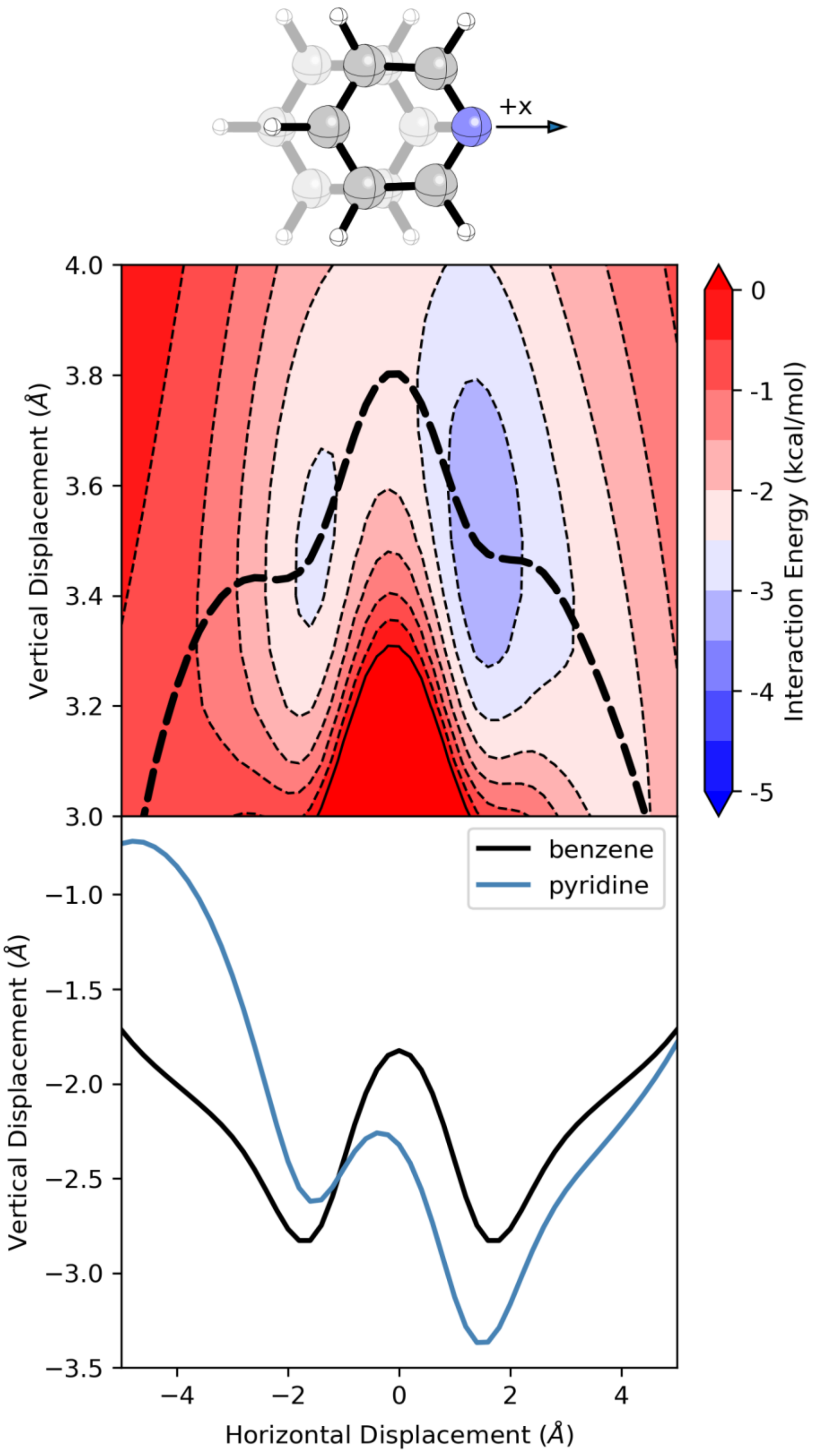

Figure S2. (top) SAPT interaction energy surface for as a function of horizontal and vertical displacements for the parallel-stacked pyridine -benzene dimer. The bold dashed line denotes the minimum energy path (MEP). (bottom) SAPT interaction energies along the corresponding MEPs for benzene-benzene and pyridine -benzene.

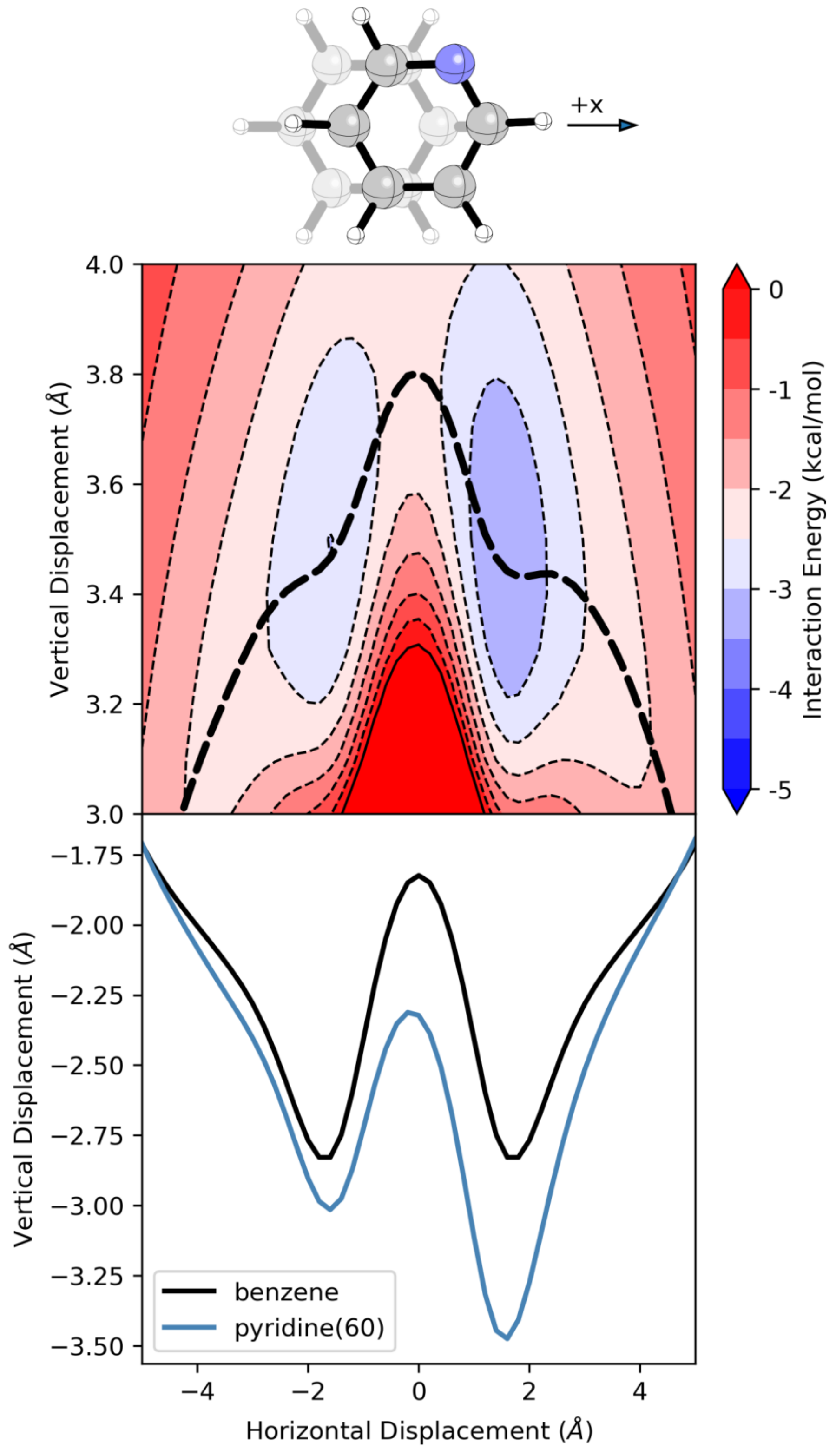

Figure S3. (top) SAPT interaction energy surface for as a function of horizontal and vertical displacements for the parallel-stacked pyridine(60)-benzene dimer. The bold dashed line denotes the minimum energy path (MEP). (bottom) SAPT interaction energies along the corresponding MEPs for benzene-benzene and pyridine(60)-benzene.

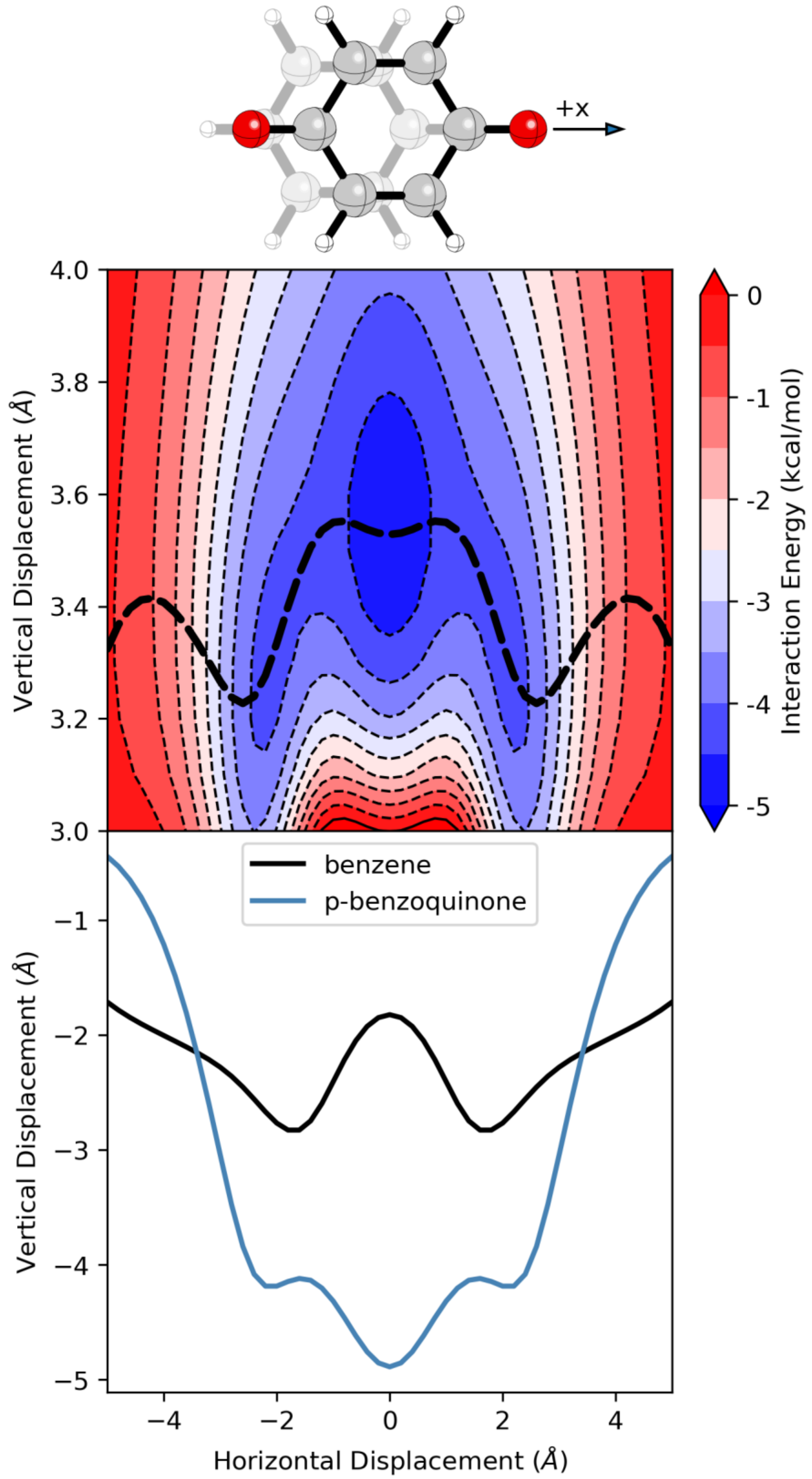

Figure S4. (top) SAPT interaction energy surface for as a function of horizontal and vertical displacements for the parallel-stacked p-benzoquinone-benzene dimer. The bold dashed line denotes the minimum energy path (MEP). (bottom) SAPT interaction energies along the corresponding MEPs for benzene-benzene and p-benzoquinone-benzene.

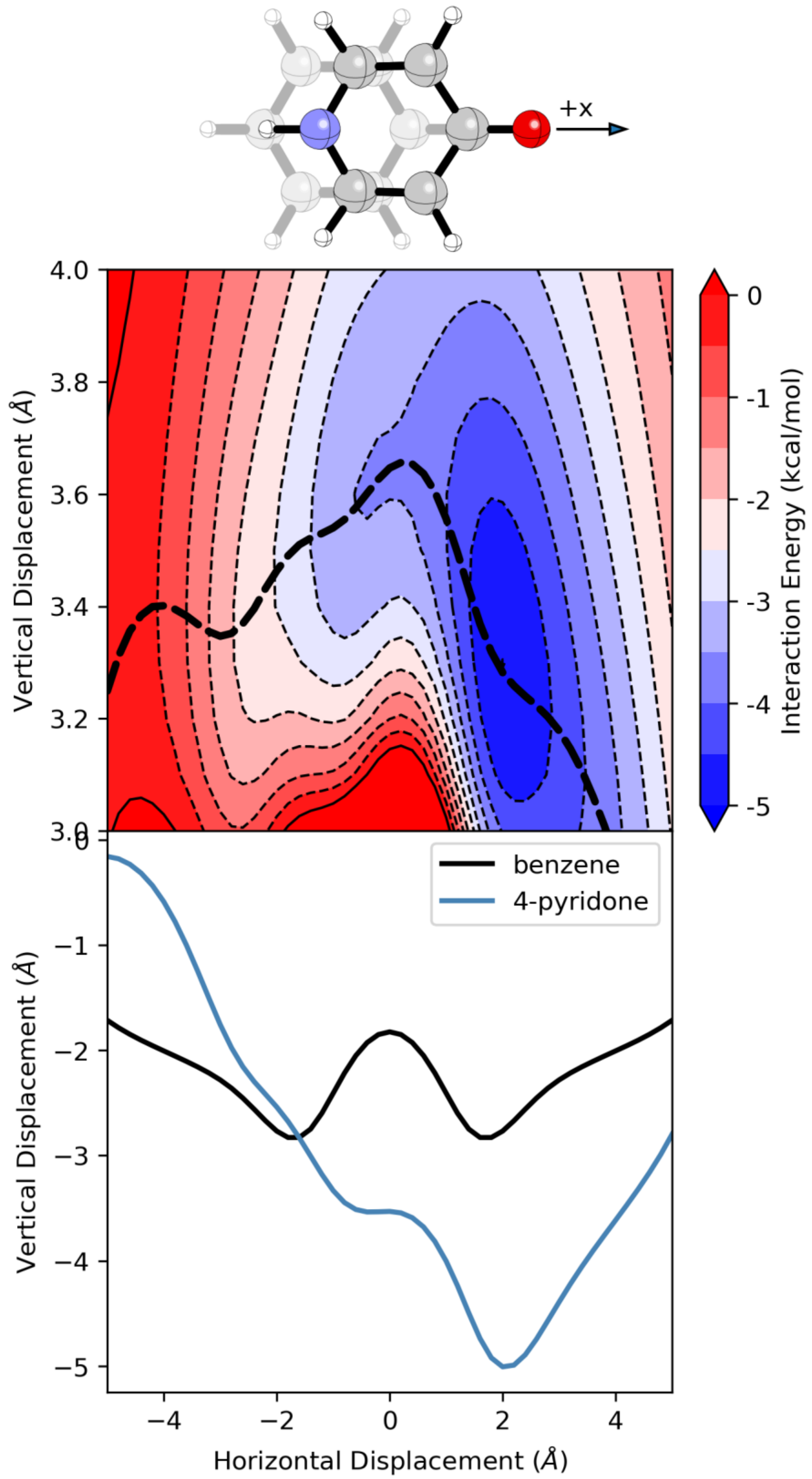

Figure S5. (top) SAPT interaction energy surface for as a function of horizontal and vertical displacements for the parallel-stacked 4-pyridone -benzene dimer. The bold dashed line denotes the minimum energy path (MEP). (bottom) SAPT interaction energies along the corresponding MEPs for benzene-benzene and 4-pyridone-benzene.

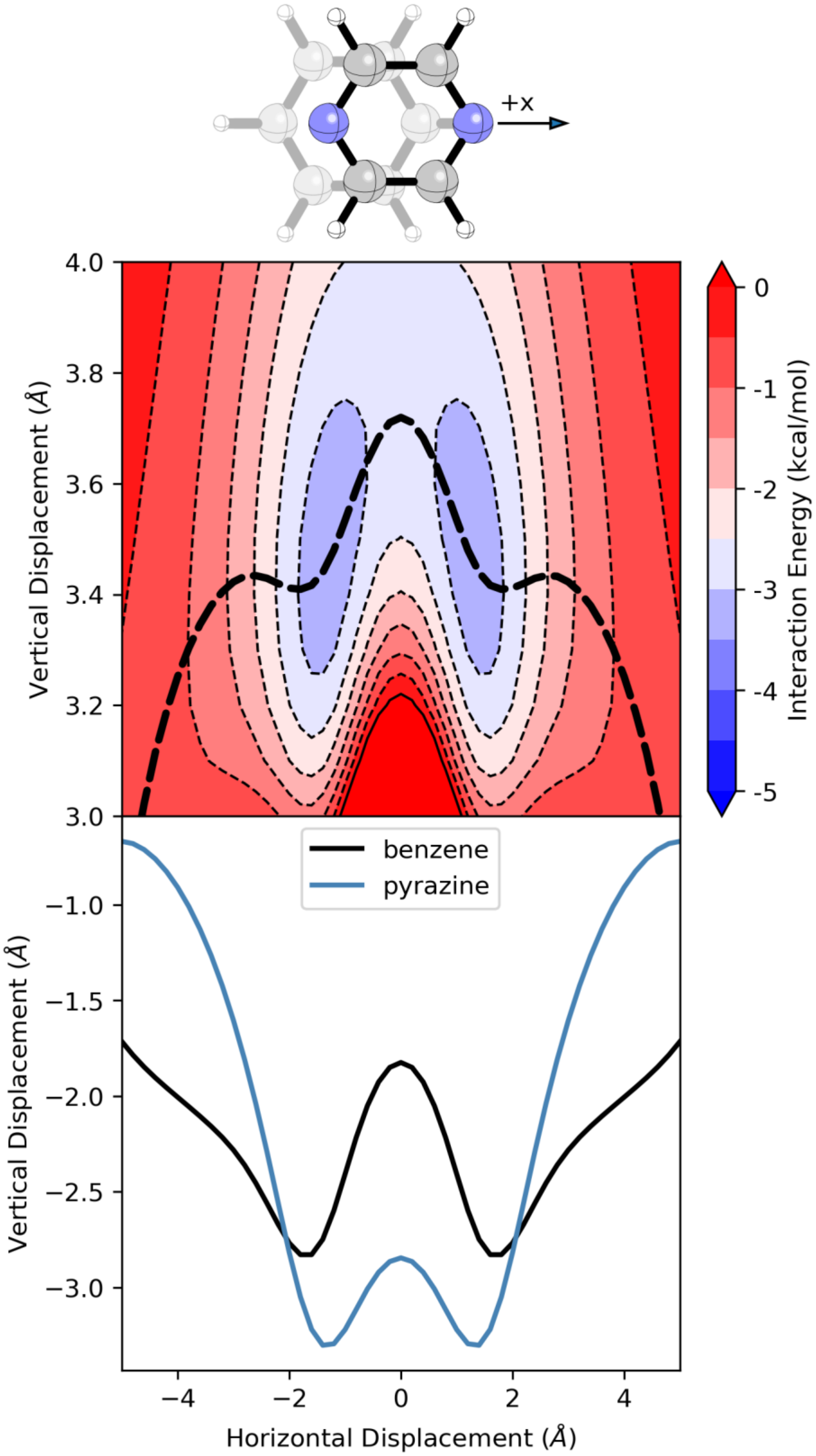

Figure S6. (top) SAPT interaction energy surface for as a function of horizontal and vertical displacements for the parallel-stacked pyrazine-benzene dimer. The bold dashed line denotes the minimum energy path (MEP). (bottom) SAPT interaction energies along the corresponding MEPs for benzene-benzene and pyrazine-benzene.

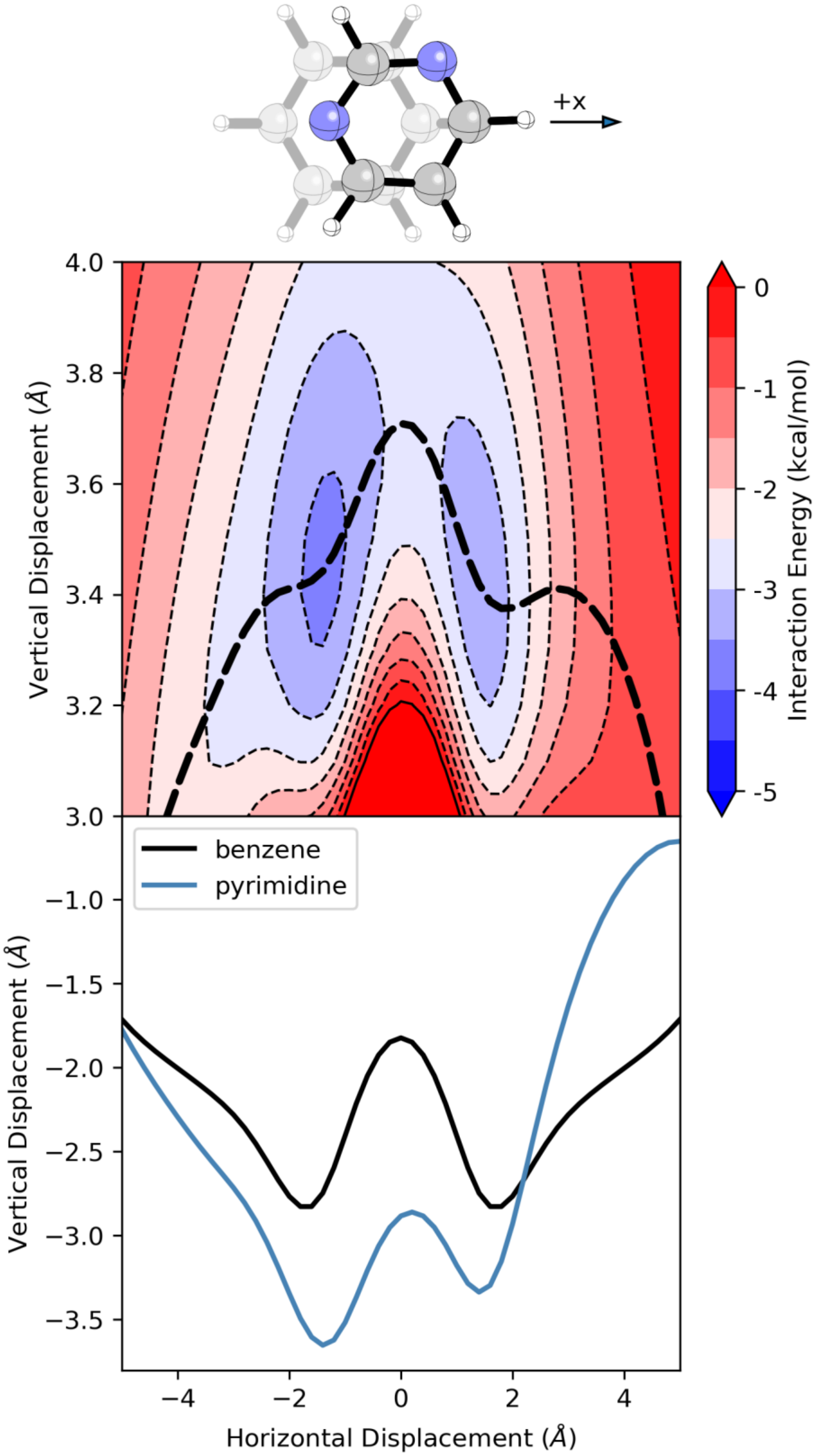

Figure S7. (top) SAPT interaction energy surface for as a function of horizontal and vertical displacements for the parallel-stacked pyrimidine-benzene dimer. The bold dashed line denotes the minimum energy path (MEP). (bottom) SAPT interaction energies along the corresponding MEPs for benzene-benzene and pyrimidine-benzene.

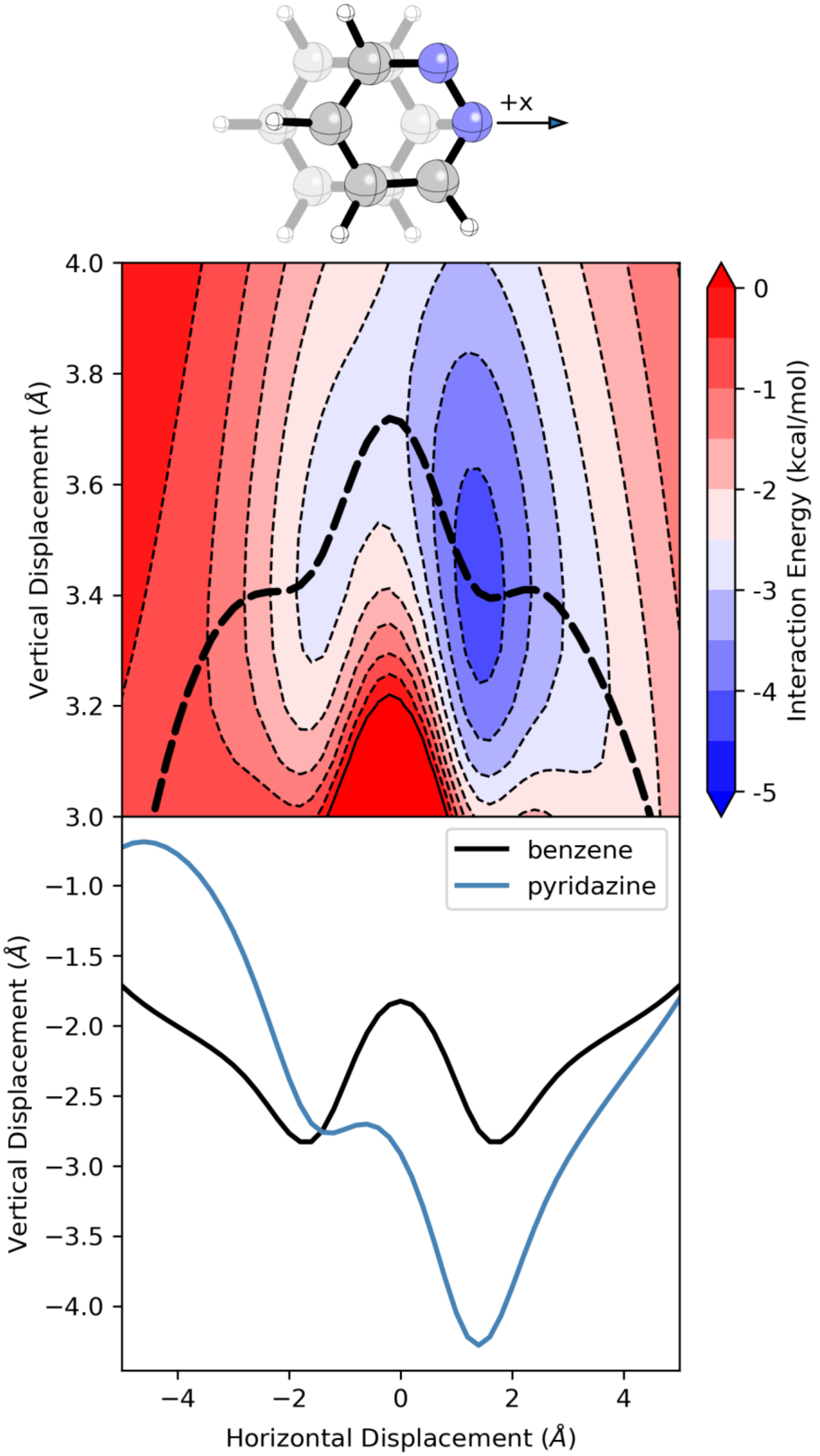

Figure S8. (top) SAPT interaction energy surface for as a function of horizontal and vertical displacements for the parallel-stacked pyridazine-benzene dimer. The bold dashed line denotes the minimum energy path (MEP). (bottom) SAPT interaction energies along the corresponding MEPs for benzene-benzene and pyridazine -benzene.

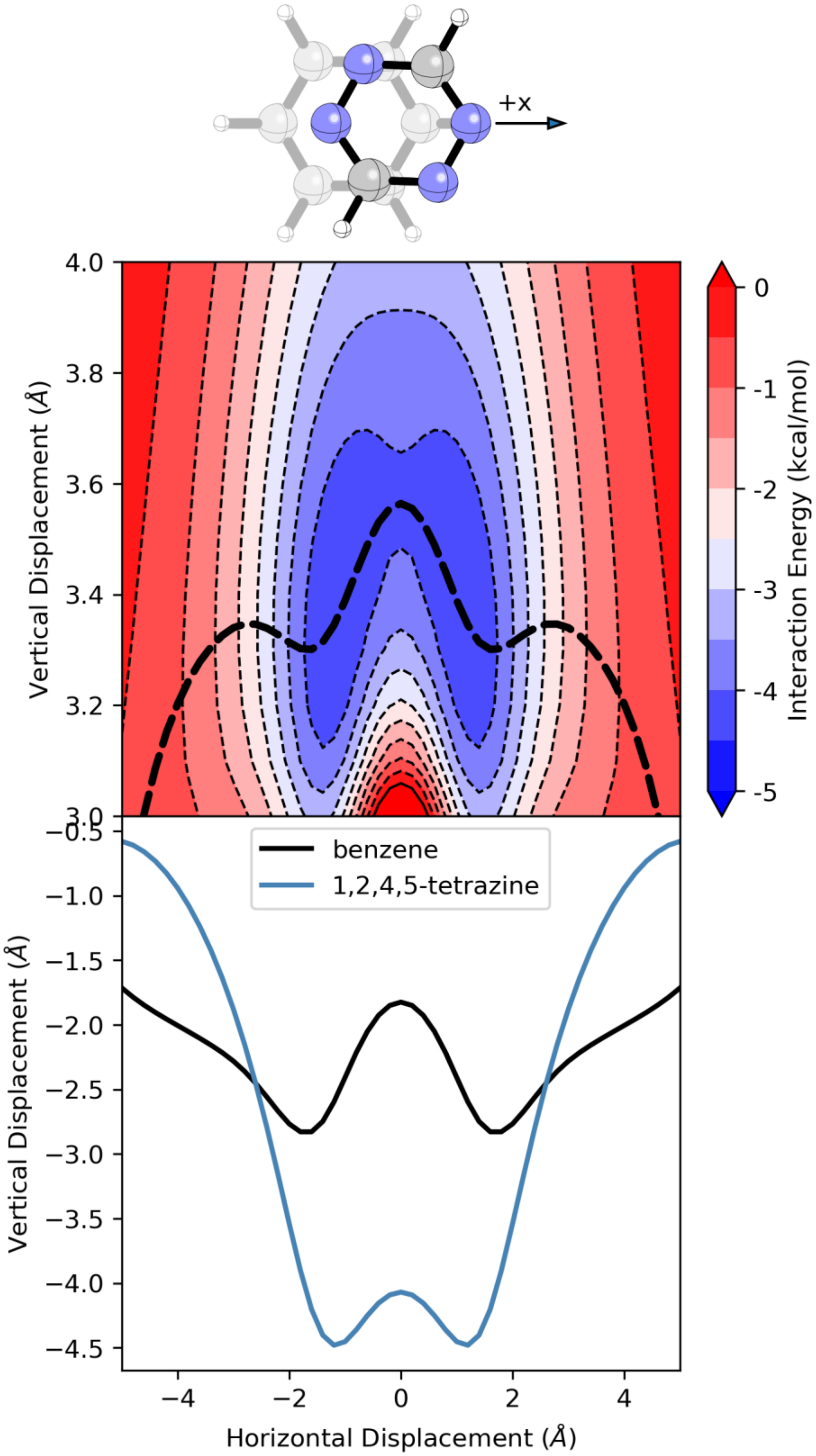

Figure S9. (top) SAPT interaction energy surface for as a function of horizontal and vertical displacements for the parallel-stacked 1,2,4,5-tetrazine-benzene dimer. The bold dashed line denotes the minimum energy path (MEP). (bottom) SAPT interaction energies along the corresponding MEPs for benzene-benzene and 1,2,4,5-tetrazine-benzene.

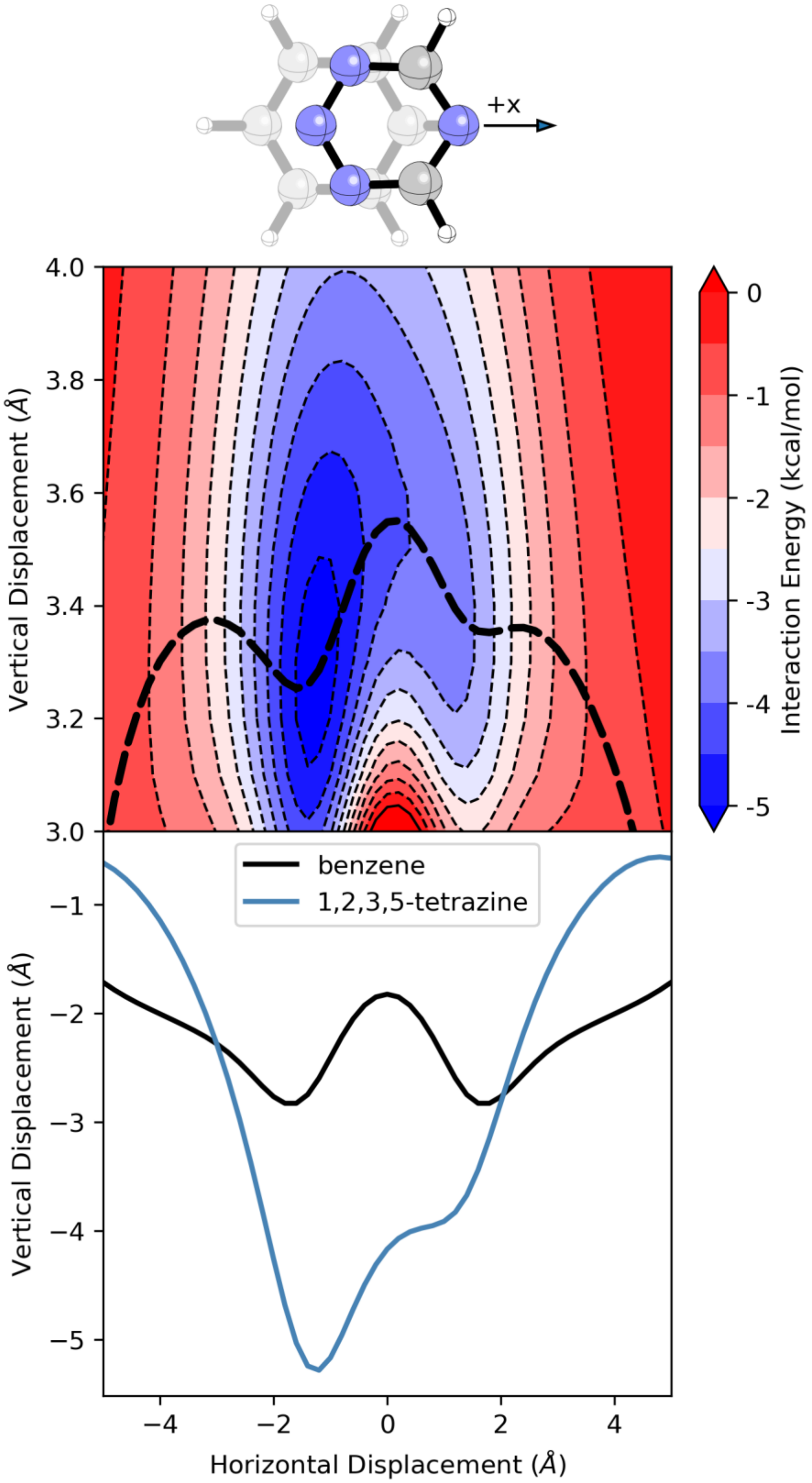

Figure S10. (top) SAPT interaction energy surface for as a function of horizontal and vertical displacements for the parallel-stacked 1,2,3,5-tetrazine -benzene dimer. The bold dashed line denotes the minimum energy path (MEP). (bottom) SAPT interaction energies along the corresponding MEPs for benzene-benzene and 1,2,3,5-tetrazine-benzene.

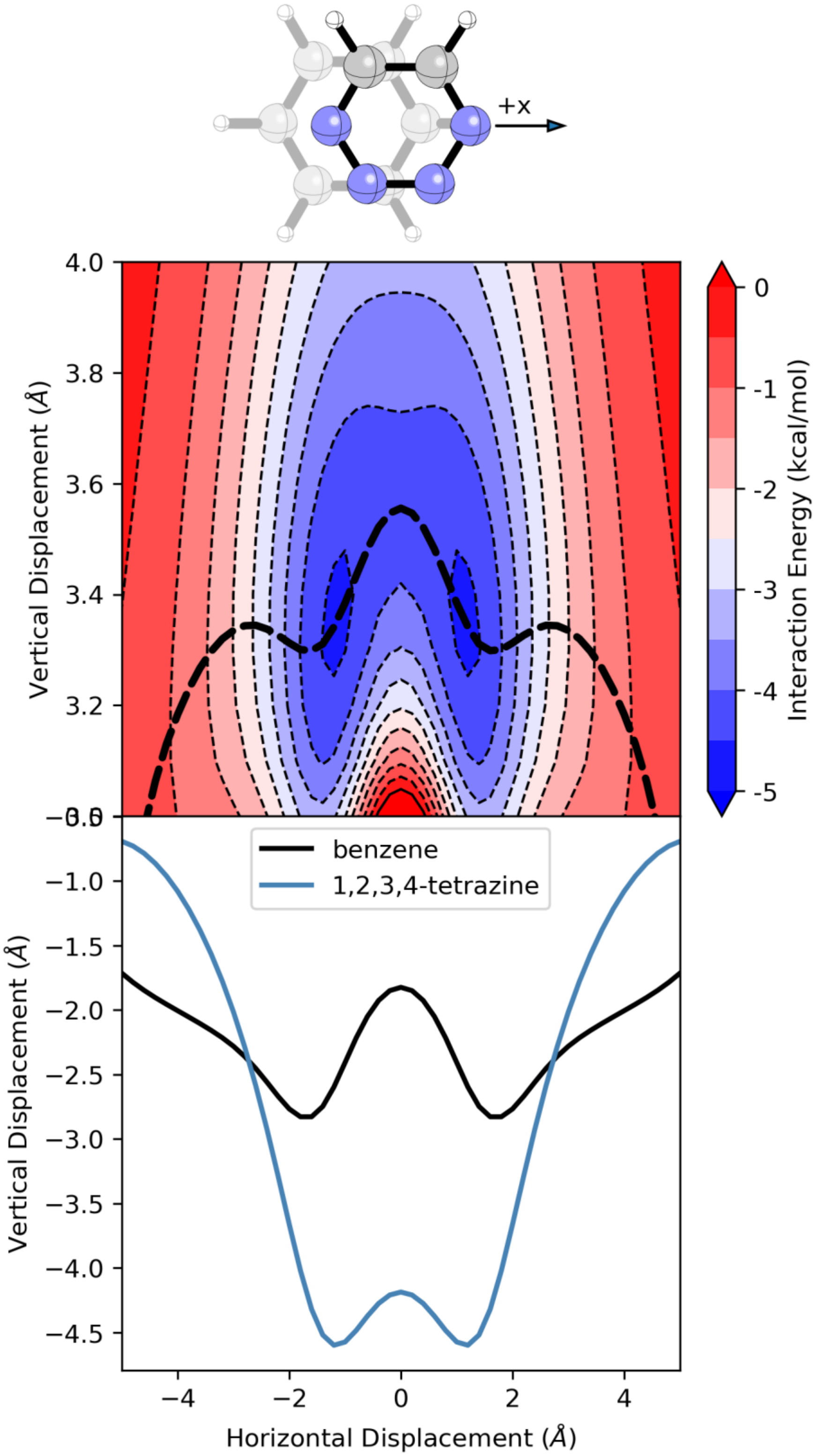

Figure S11. (top) SAPT interaction energy surface for as a function of horizontal and vertical displacements for the parallel-stacked 1,2,3,4-tetrazine -benzene dimer. The bold dashed line denotes the minimum energy path (MEP). (bottom) SAPT interaction energies along the corresponding MEPs for benzene-benzene and 1,2,3,4-tetrazine -benzene.

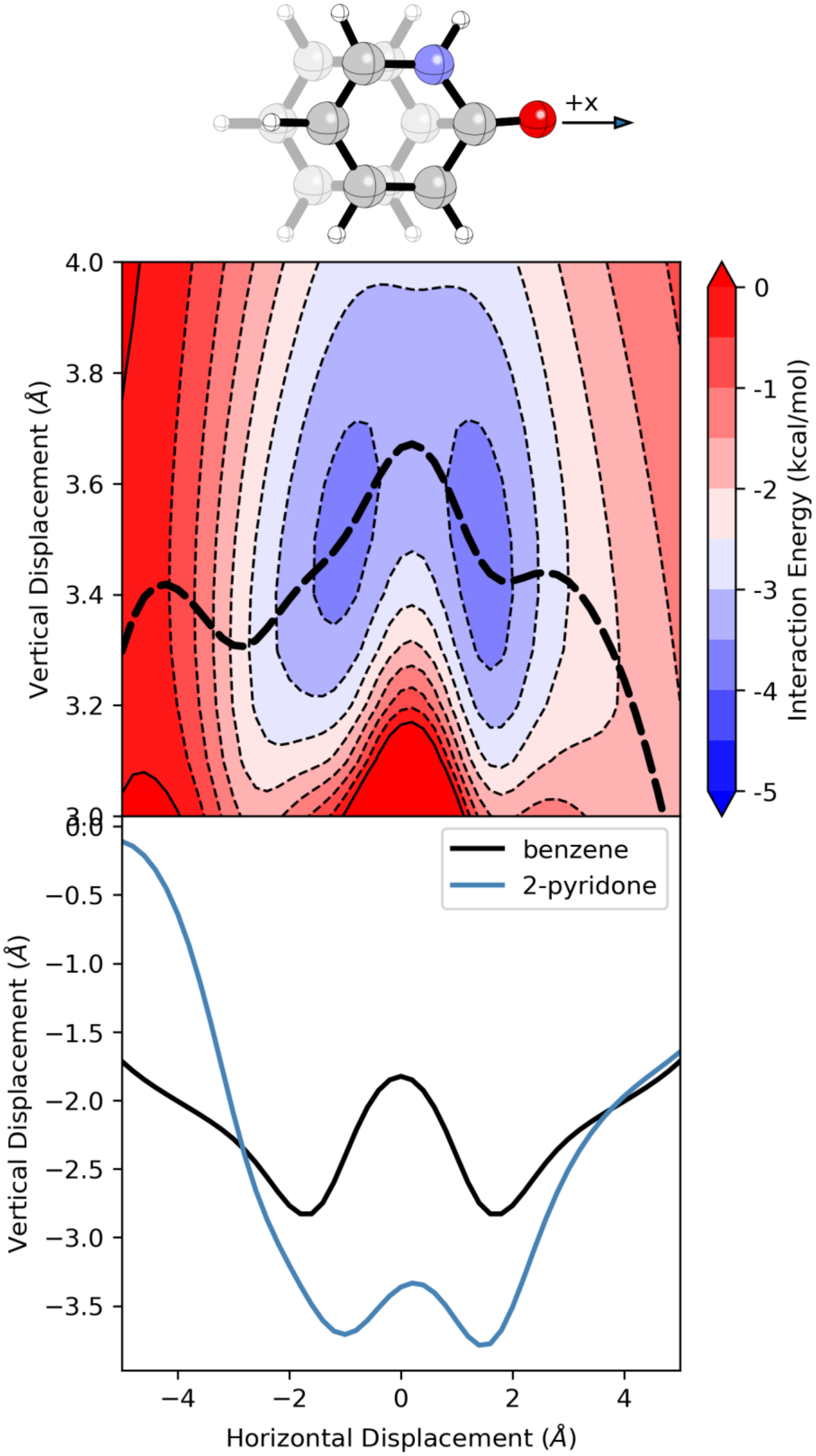

Figure S12. (top) SAPT interaction energy surface for as a function of horizontal and vertical displacements for the parallel-stacked 2-pyridone -benzene dimer. The bold dashed line denotes the minimum energy path (MEP). (bottom) SAPT interaction energies along the corresponding MEPs for benzene-benzene and 2-pyridone-benzene.

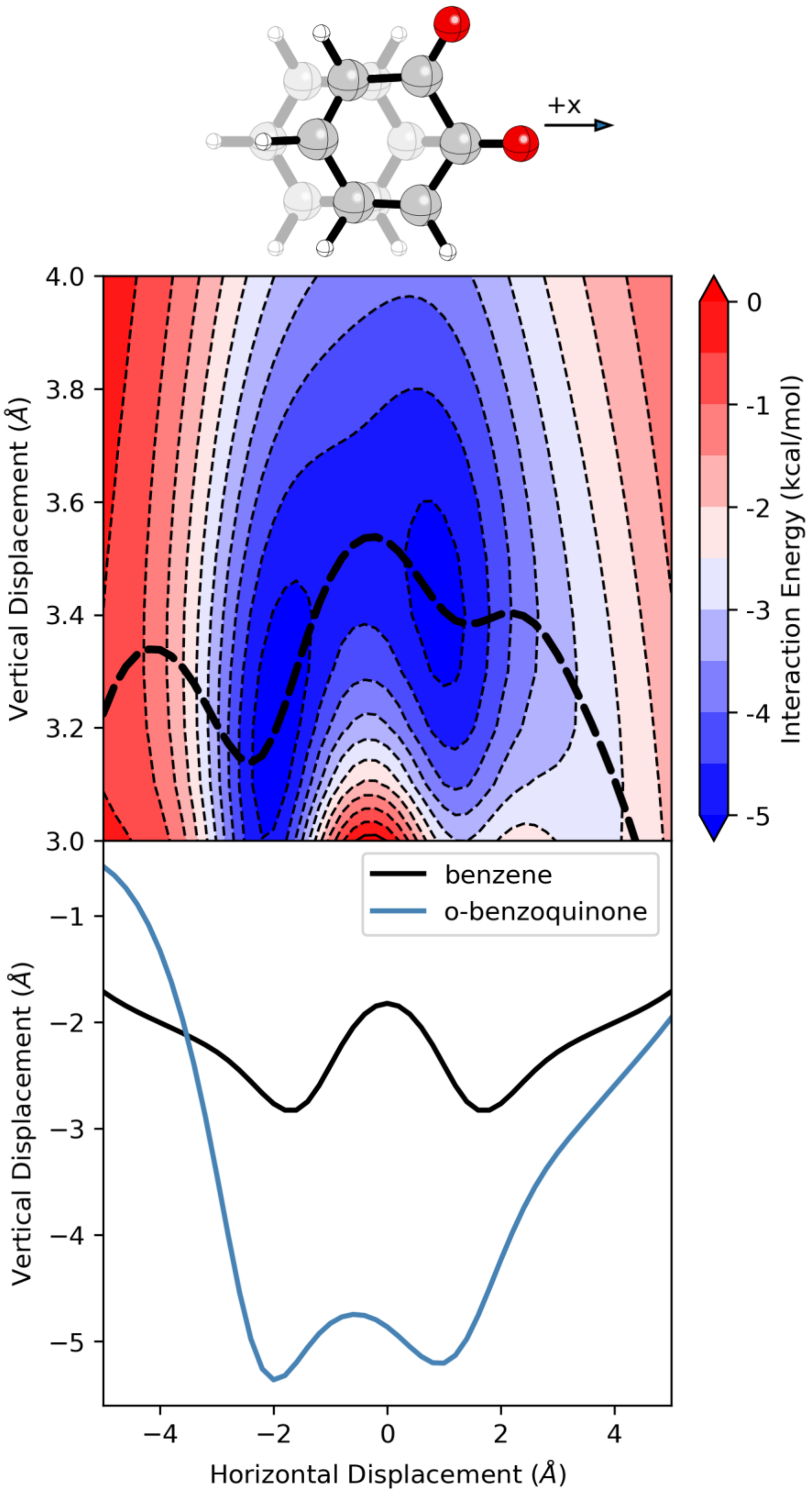

Figure S13. (top) SAPT interaction energy surface for as a function of horizontal and vertical displacements for the parallel-stacked o-benzoquinone-benzene dimer. The bold dashed line denotes the minimum energy path (MEP). (bottom) SAPT interaction energies along the corresponding MEPs for benzene-benzene and o-benzoquinone-benzene.

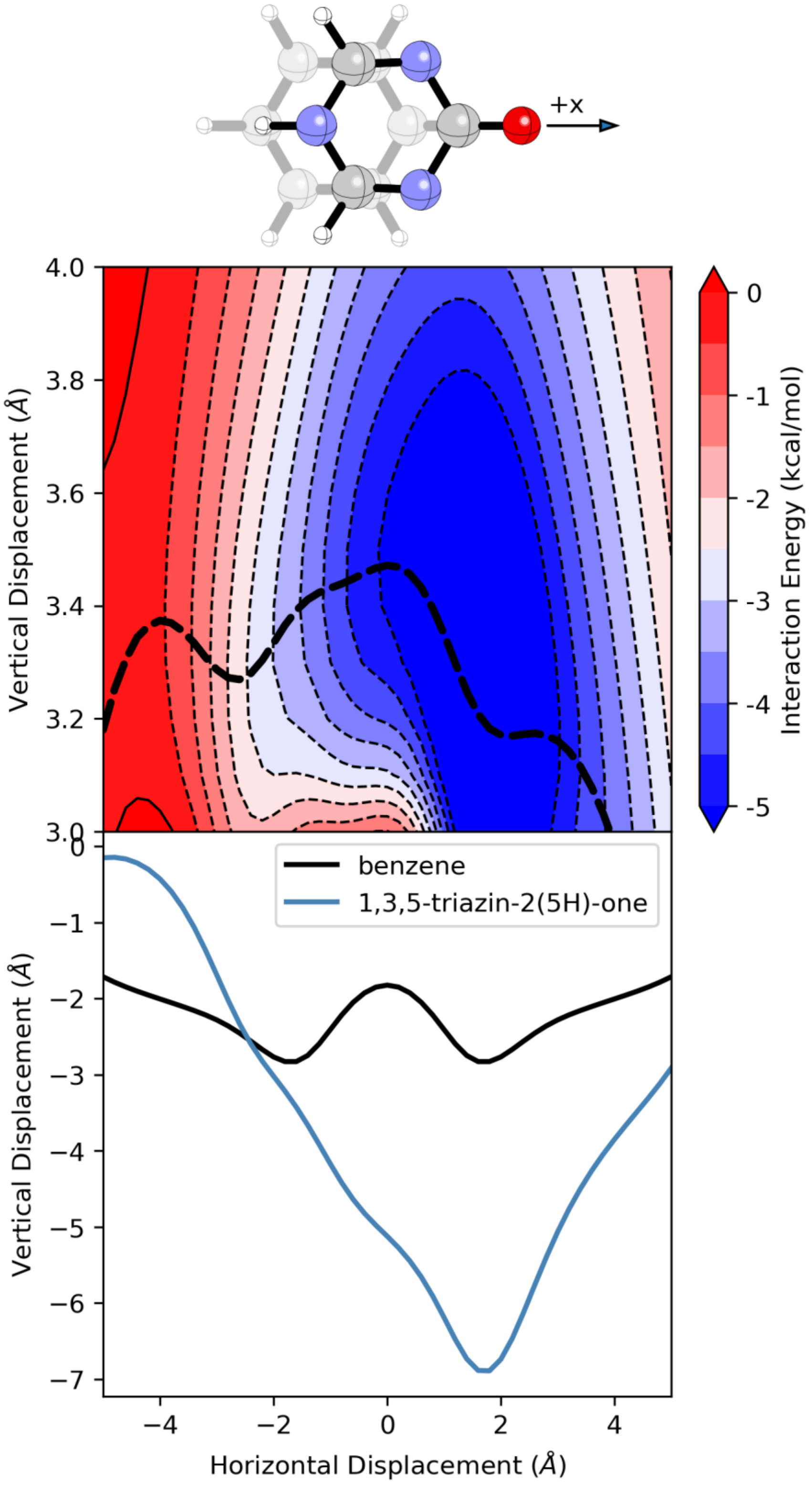

Figure S14. (top) SAPT interaction energy surface for as a function of horizontal and vertical displacements for the parallel-stacked 1,3,5-triazin-2(5H)-one-benzene dimer. The bold dashed line denotes the minimum energy path (MEP). (bottom) SAPT interaction energies along the corresponding MEPs for benzene-benzene and 1,3,5-triazin-2(5H)-one-benzene.

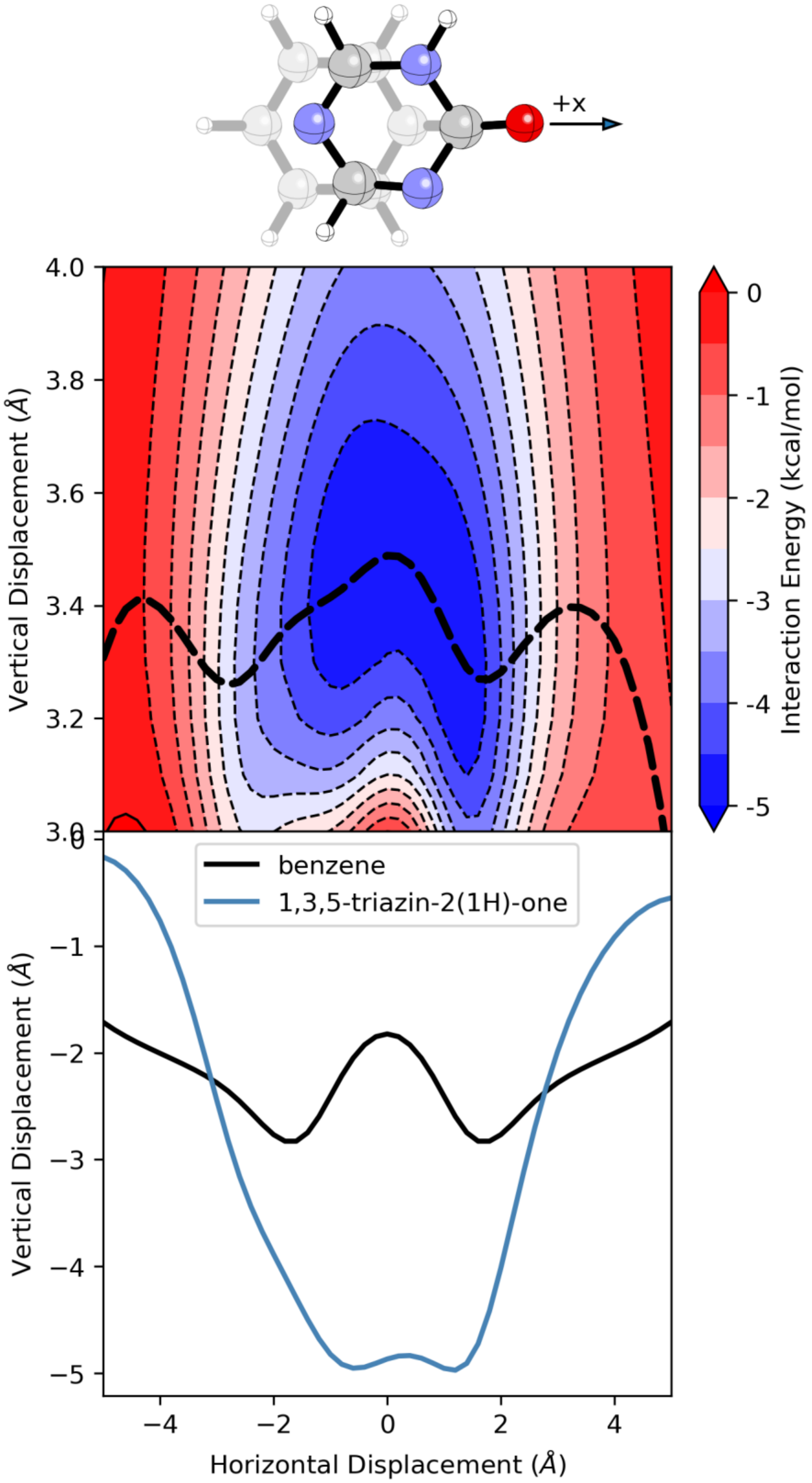

Figure S15. (top) SAPT interaction energy surface for as a function of horizontal and vertical displacements for the parallel-stacked 1,3,5-triazin-2(1H)-one-benzene dimer. The bold dashed line denotes the minimum energy path (MEP). (bottom) SAPT interaction energies along the corresponding MEPs for benzene-benzene and 1,3,5-triazin-2(1H)-one-benzene.

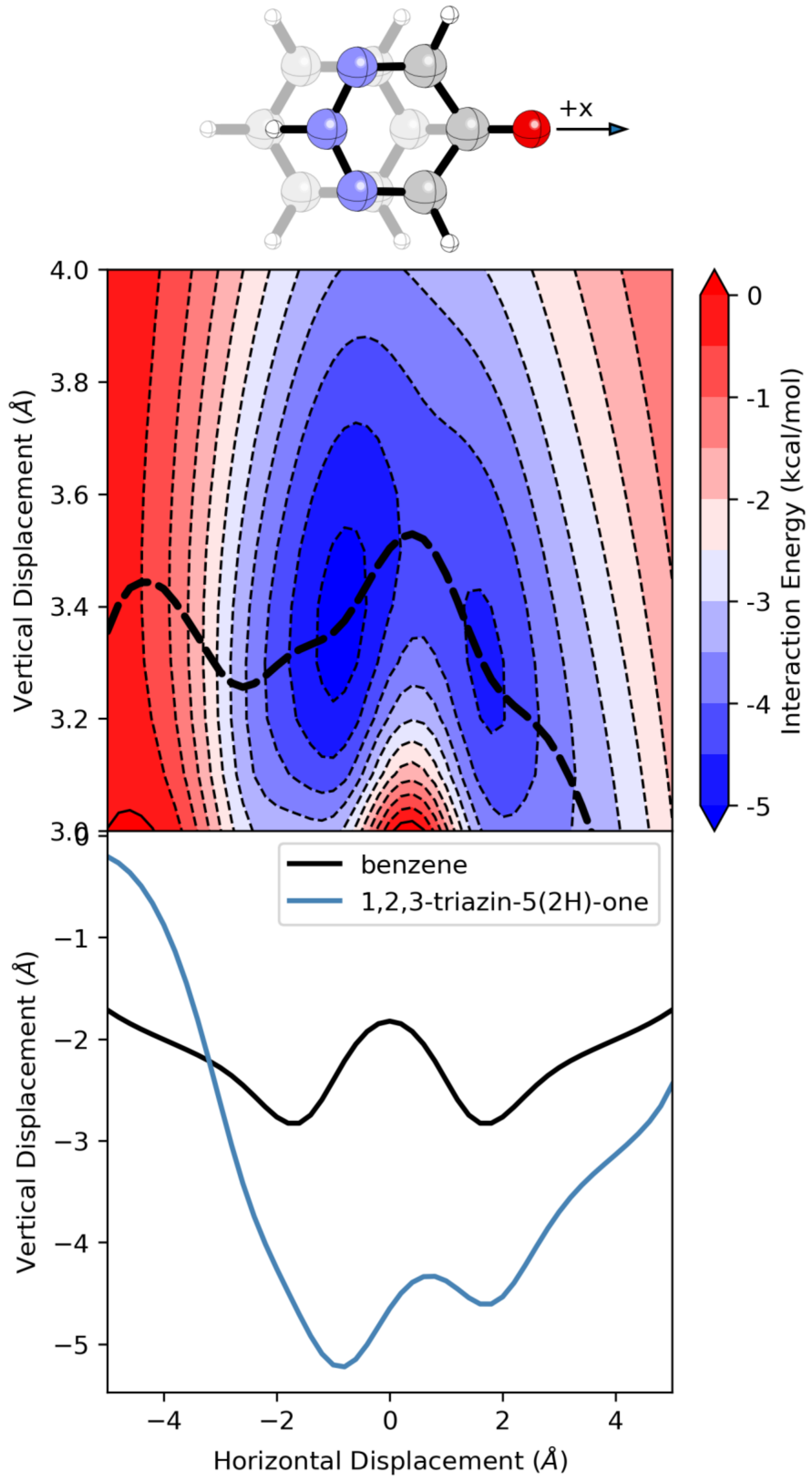

Figure S16. (top) SAPT interaction energy surface for as a function of horizontal and vertical displacements for the parallel-stacked 1,2,3-triazin-5(2H)-one-benzene dimer. The bold dashed line denotes the minimum energy path (MEP). (bottom) SAPT interaction energies along the corresponding MEPs for benzene-benzene and 1,2,3-triazin-5(2H)-one-benzene.

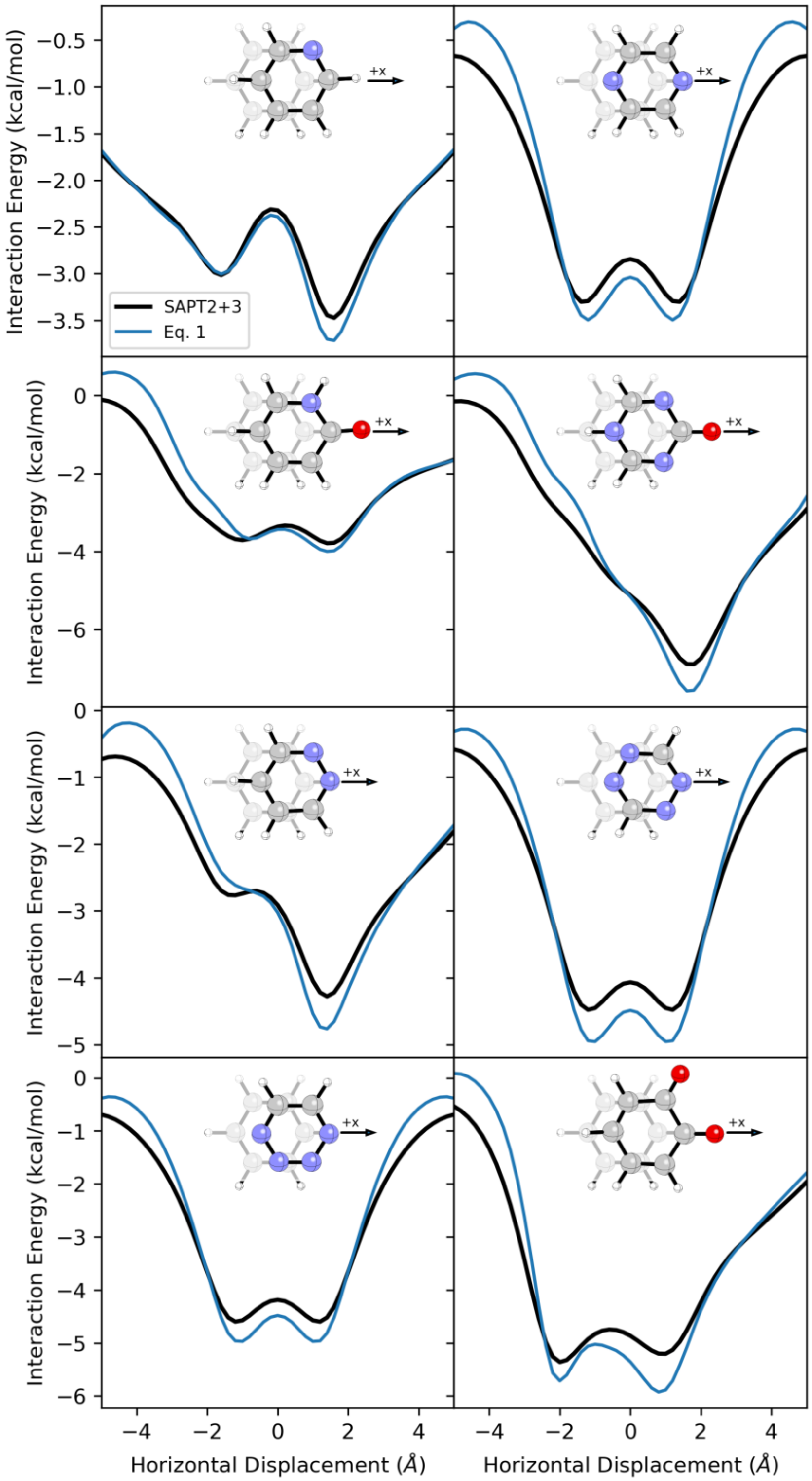

Figure S17. SAPT interaction energy curves (black) for dimers of benzene with selected heterocycles as well as approximate energy curves (blue) from Eq. 1 from the main text.

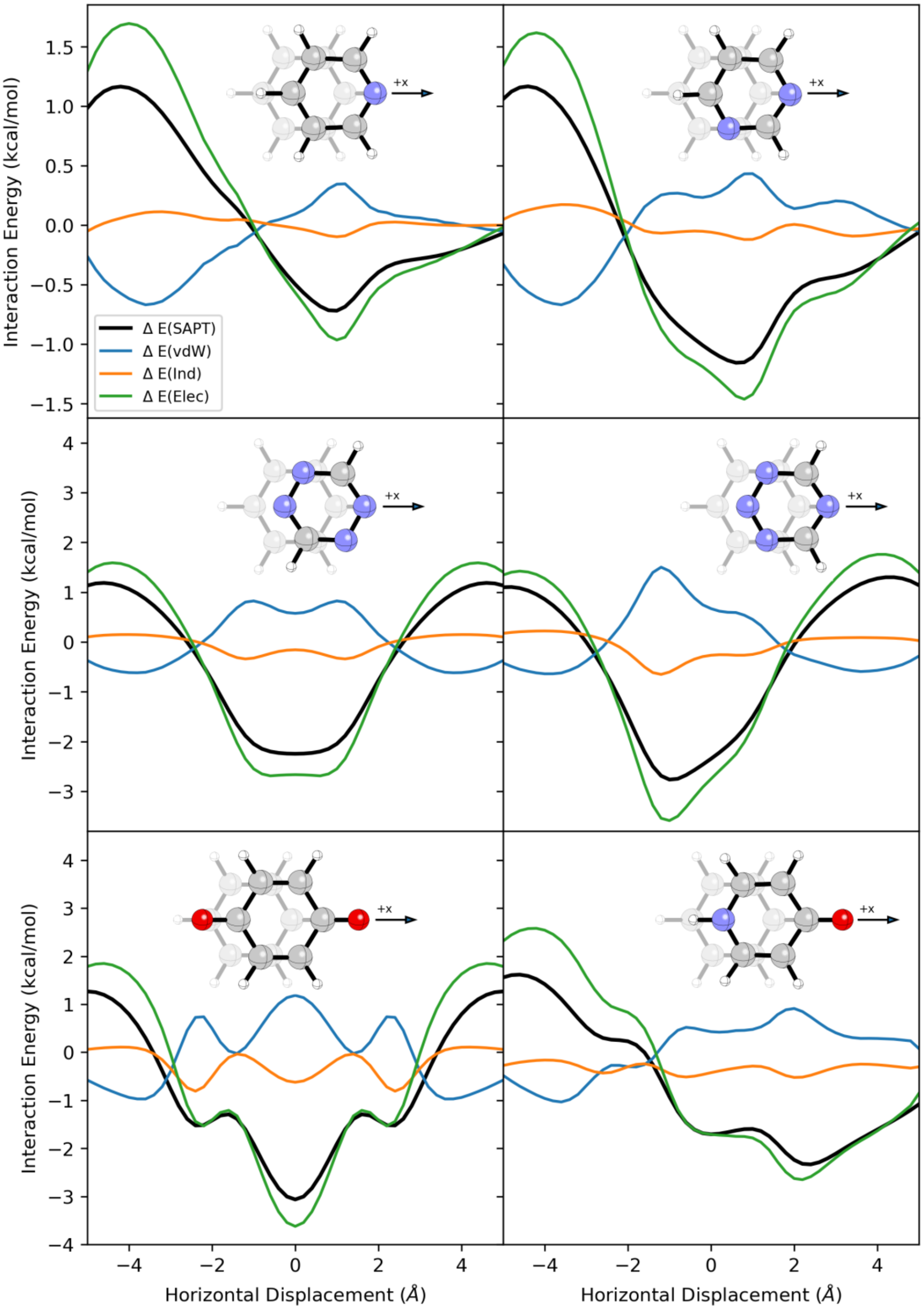

Figure S18. Difference in the SAPT interaction energy [ΔE(SAPT)] as well as components between the pictured benzene-heterocyle dimer and the benzene-benzene dimer along the corresponding MEPs.

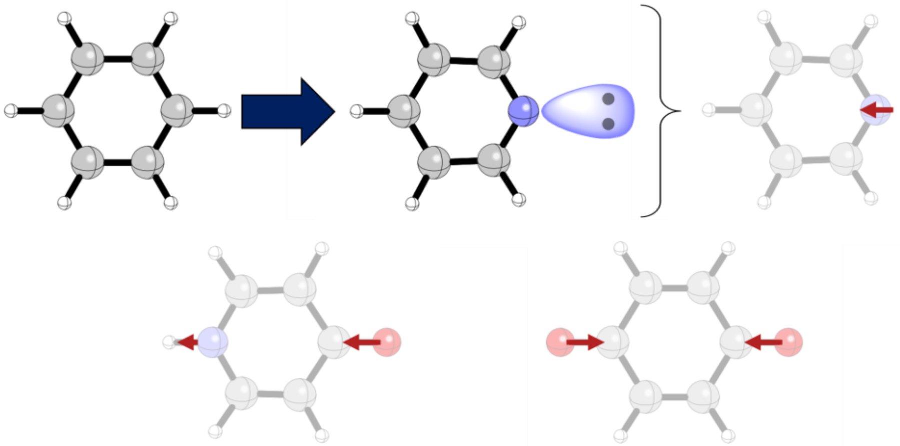

Figure S19. The impact of replacing CH with N: on stacking interactions can be captured by a radial point dipole directed toward the ring centroid and located at the position of the N atom. The effect of NH and CO groups can also be approximately captured by appropriately oriented local dipoles.

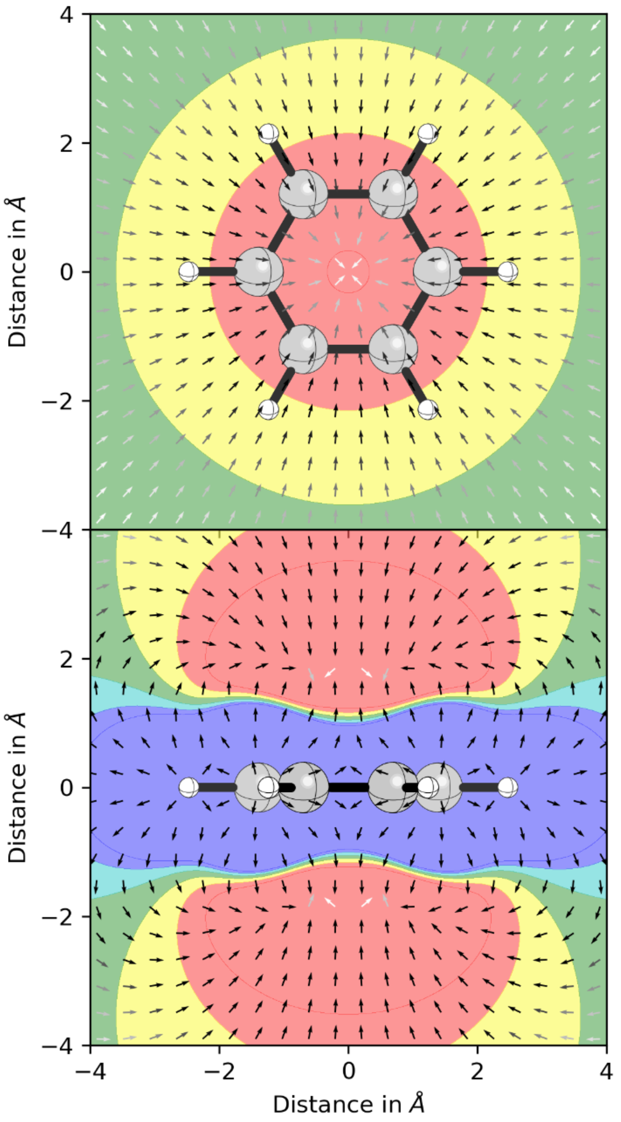

Figure S20. Electrostatic potential of benzene and component of the electric field (arrows, with darker color corresponding to stronger field) in the plane (top) 3.5 Å from the molecular plane and (bottom) bisecting the ring.

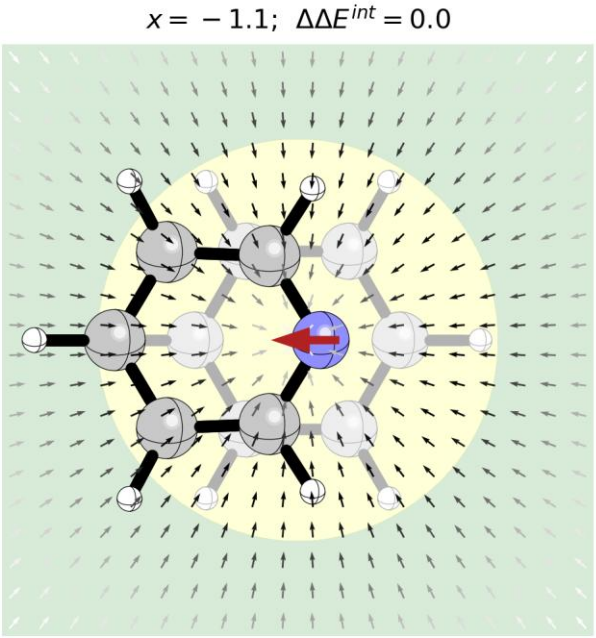

Figure S21. Parallel stacked dimer of benzene with pyridine at $x = -1.1$ Å along with the ESP and electric field of benzene. Interaction energy ($\Delta\Delta E^{int}$), relative to the benzene dimer at the corresponding horizontal displacement given in kcal/mol. Red arrow denotes the local dipole representing the N atom in pyridine.

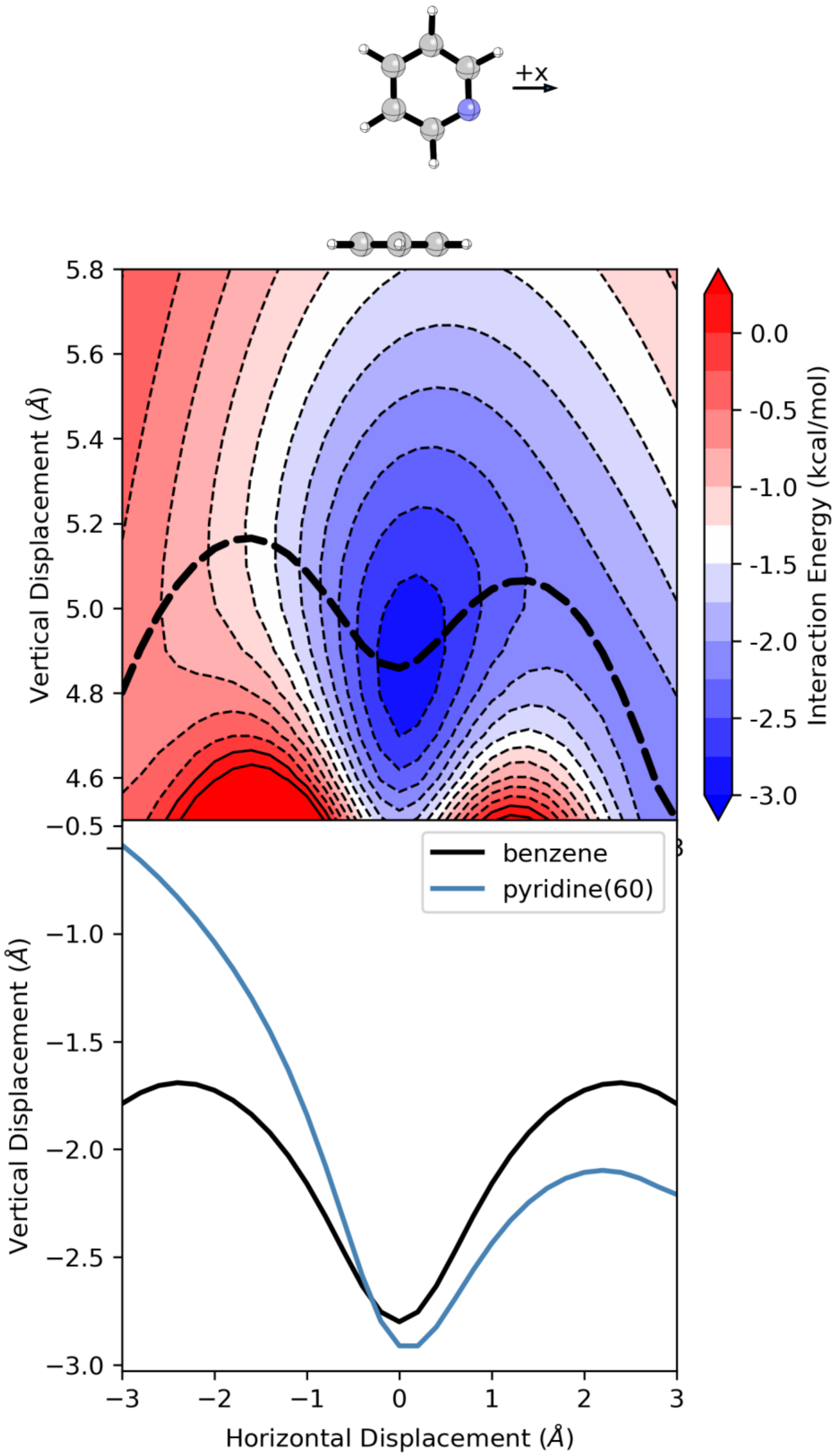

Figure S22. (top) SAPT interaction energy surface for as a function of horizontal and vertical displacements for the T-shaped pyridine(60)-benzene dimer. The bold dashed line denotes the minimum energy path (MEP). (bottom) SAPT interaction energies along the corresponding MEPs for benzene-benzene and pyridine(60)-benzene.

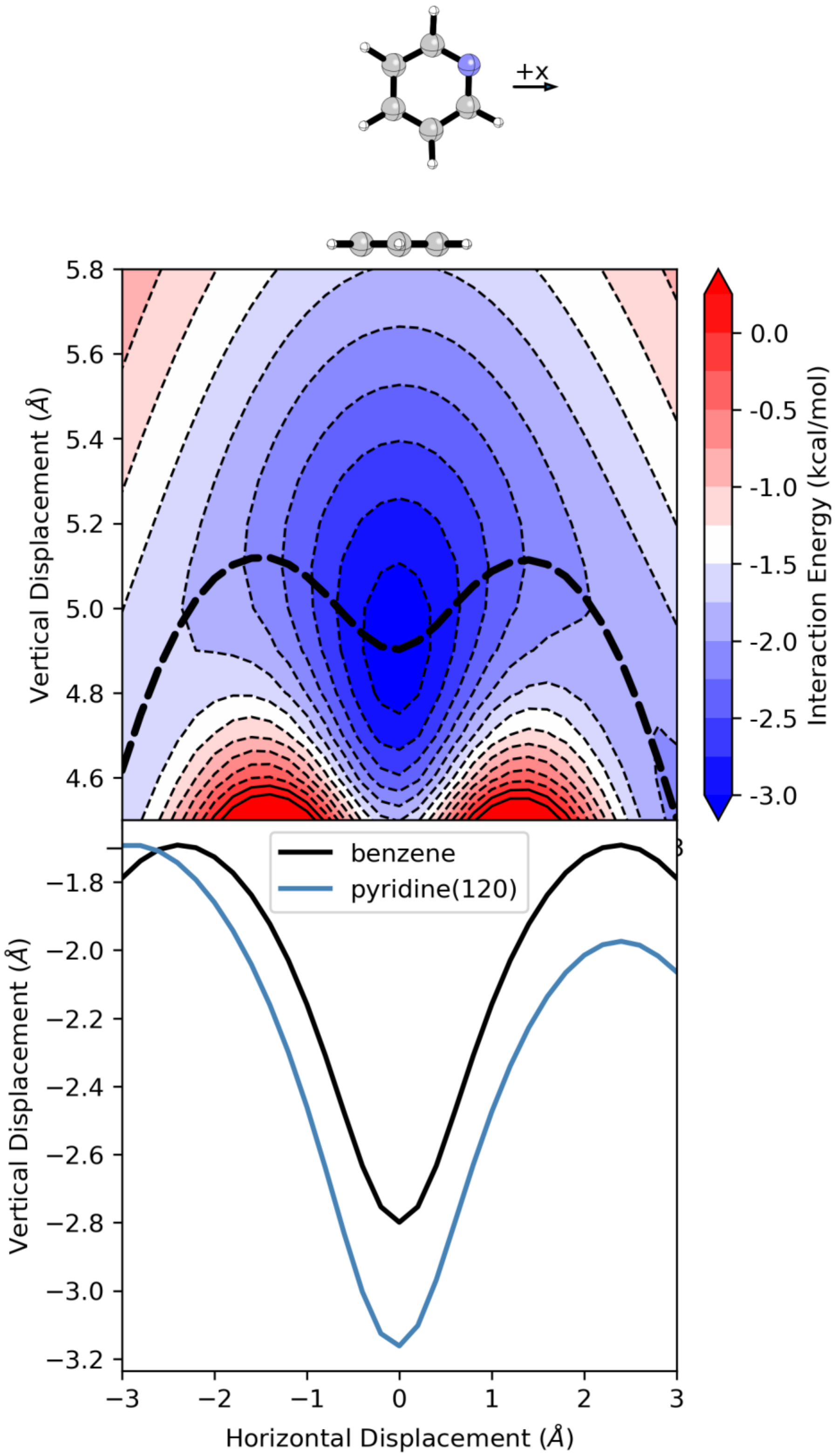

Figure S23. (top) SAPT interaction energy surface for as a function of horizontal and vertical displacements for the T-shaped pyridine(120)-benzene dimer. The bold dashed line denotes the minimum energy path (MEP). (bottom) SAPT interaction energies along the corresponding MEPs for benzene-benzene and pyridine(120)-benzene.

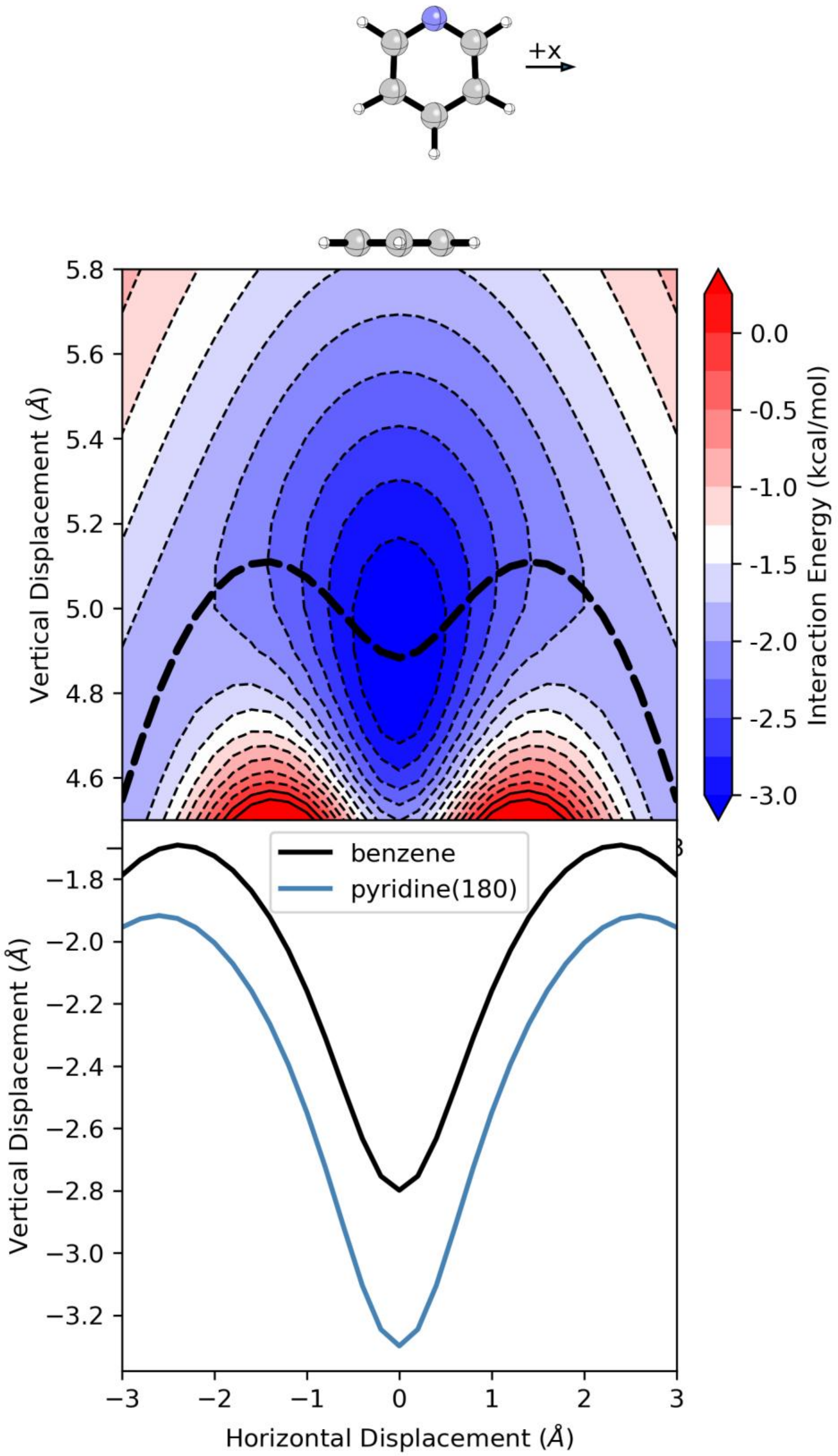

Figure S24. (top) SAPT interaction energy surface for as a function of horizontal and vertical displacements for the T-shaped pyridine(180)-benzene dimer. The bold dashed line denotes the minimum energy path (MEP). (bottom) SAPT interaction energies along the corresponding MEPs for benzene-benzene and pyridine(180)-benzene.

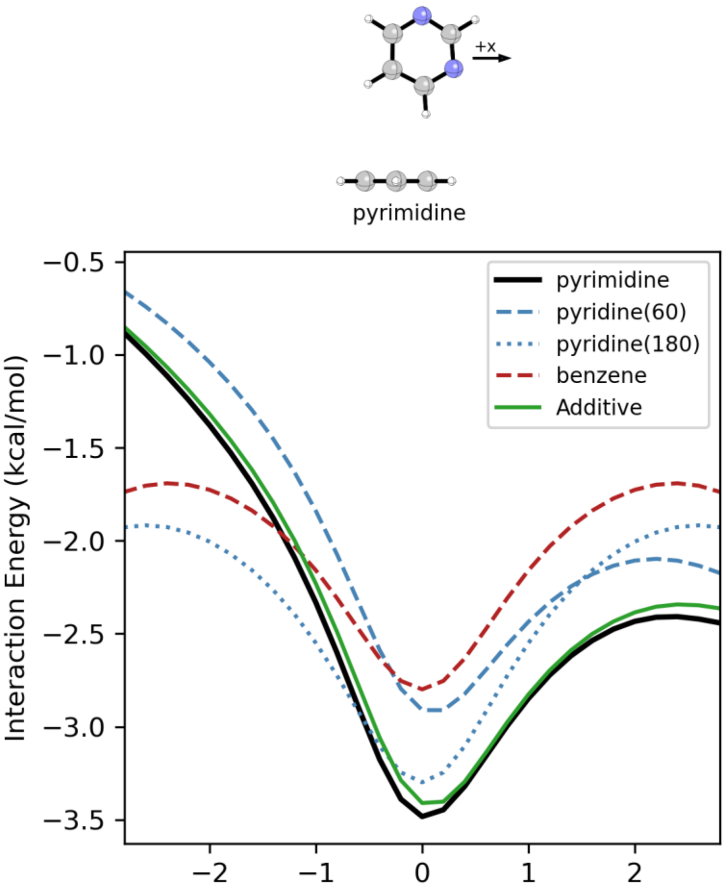

Figure S25. SAPT interaction energy curves for the T-shaped dimer of pyrimidine and benzene (black) along with additive approximation (green) arising from the sum of the blue energy curves minus the red energy curve.

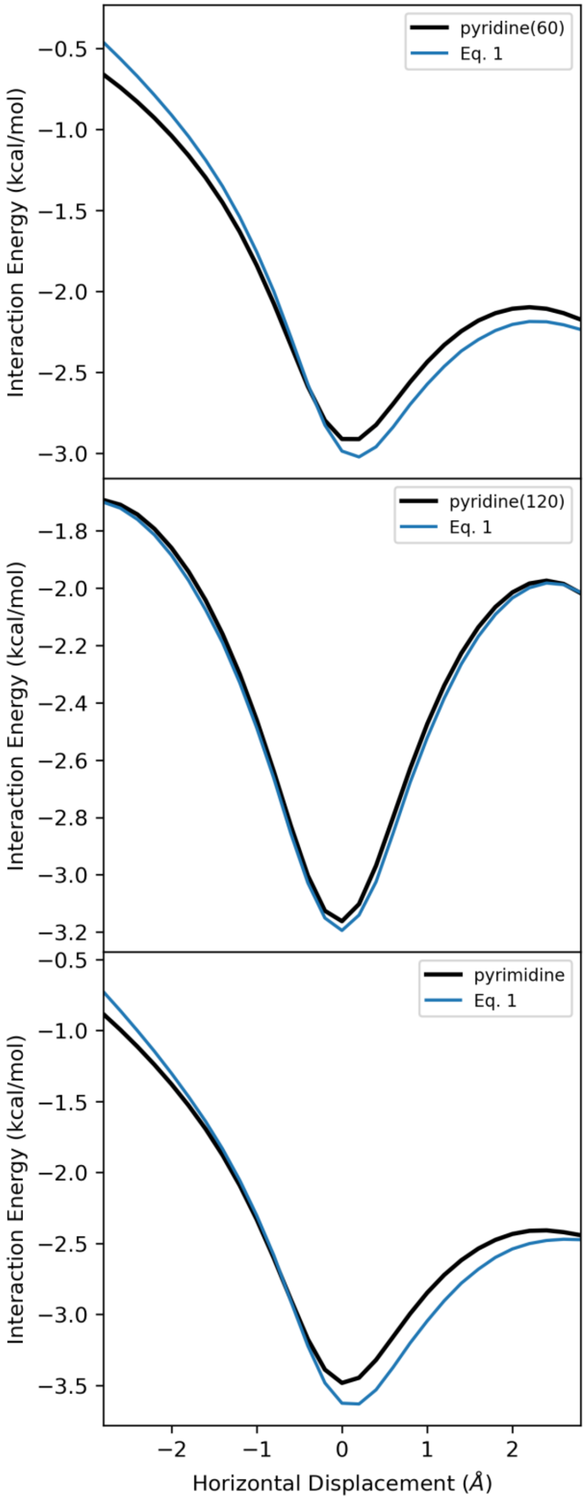

Figure S26. SAPT interaction energy curves (black) for T-shaped dimers of benzene with pyridine(60), pyridine(120) and primidine as well as approximate energy curves (blue) from Eq. 1 from the main text.

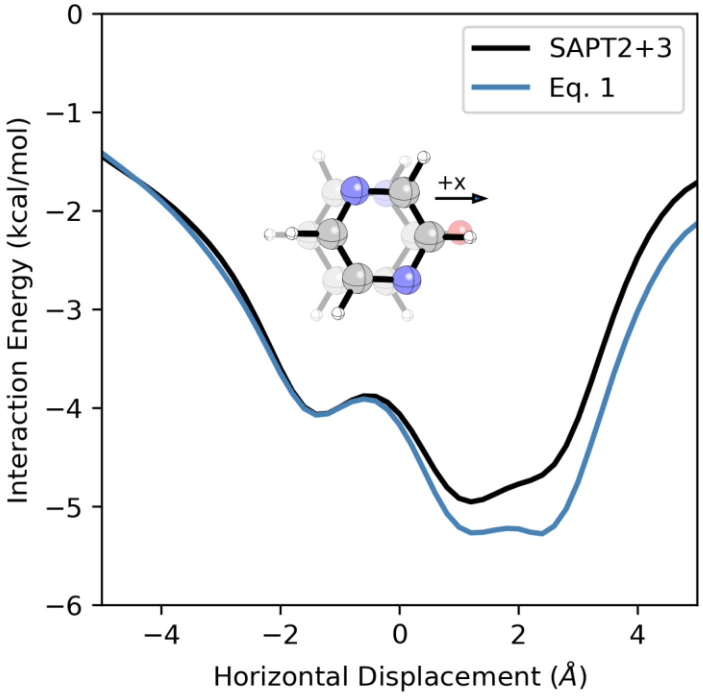

Figure S27. SAPT interaction energy curves (black) for parallel stacked dimer of pyrazine(120) with 2-pyridone as well as approximate energy curves (blue) from Eq. 1 from the main text.

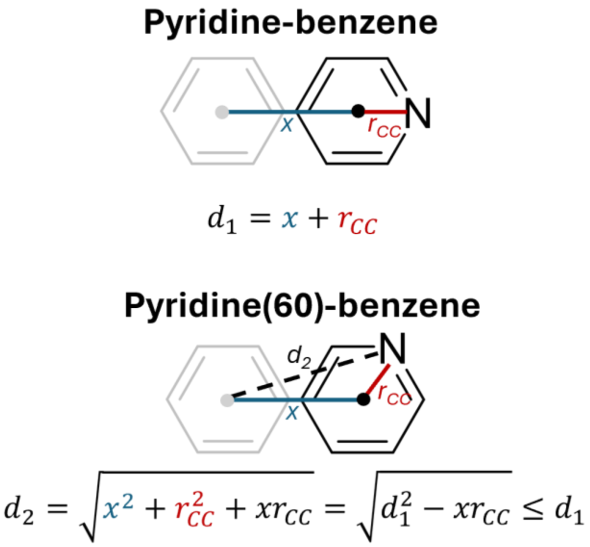

Figure S28. Distances between the ring centroid of benzene and the N atom in pyridine (d_1) and pyridine(60) (d_2) in dimers displaced along the +*x* axis under the assumption that pyridine is a regular hexagon with side r_{CC}.

References

(1) Hohenstein, E. G.; Sherrill, C. D. Density Fitting of Intramonomer Correlation Effects in Symmetry-Adapted Perturbation Theory *J. Chem. Phys.* **2010**, *133*, 014101.
(2) Hohenstein, E. G.; Sherrill, C. D. Wavefunction methods for noncovalent interactions. *WIREs Comp. Mol. Sci.* **2012**, *2*, 304-326.
(3) Weigend, F.; Ahlrichs, R. Balanced basis sets of split valence, triple zeta valence and quadruple zeta valence quality for H to Rn: Design and assessment of accuracy. *Phys. Chem. Chem. Phys.* **2005**, *7*, 3297-3305.
(4) Turney, J. M.; Simmonett, A. C.; Parrish, R. M.; Hohenstein, E. G.; Evangelista, F. A.; Fermann, J. T.; Mintz, B. J.; Burns, L. A.; Wilke, J. J.; Abrams, M. L.; et al. PSI4: an open-source ab initio electronic structure program. *Wires Comput Mol Sci* **2012**, *2* (4), 556-565.
(5) Wheeler, S. E. Revisiting the Hunter-Sanders Model for pi-pi Interactions. *J Am Chem Soc* **2025**, *147* (23), 19738-19750.
(6) Parker, T. M.; Burns, L. A.; Parrish, R. M.; Ryno, A. G.; Sherrill, C. D. Levels of symmetry adapted perturbation theory (SAPT). I. Efficiency and performance for interaction energies. *J. Chem. Phys.* **2014**, *140*, 094106.
(7) *Gaussian 16 Rev. C.01*; Wallingford, CT, 2016.
(8) Ingman, V. M.; Schaefer, A. J.; Andreola, L. R.; Wheeler, S. E. QChASM: Quantum chemistry automation and structure manipulation. *WIREs Computational Molecular Science* **2020**, *11* (4), e1510.
